# Stable and temporally shifted DNA methylation differences between 14-year-old Norway spruce epitypes under common garden conditions

**DOI:** 10.1007/s00425-026-05116-6

**Published:** 2026-08-07

**Authors:** Igor A. Yakovlev, Marcos Viejo, Torstein Tengs, Jorunn E. Olsen, Carl G. Fossdal

**Affiliations:** 1 Department of Plant Molecular Biology, Norwegian Institute of Bioeconomy Research, Ås, Norway; 2 Department of Functional Biology, University of Santiago de Compostela, Santiago de Compostela, Spain; 3 Department of Breeding and Genetics, Norwegian Food Research Institute, Ås, Norway; 4 Department of Plant Sciences, Faculty of Biosciences, Norwegian University of Life Sciences, Ås, Norway

**Keywords:** Bud phenology, DNA methylation, Embryogenesis, Epigenetic memory, Epitype, *Picea abies*, Promoter

## Abstract

**Main conclusion:**

Dynamic DNA methylation differences between epitypes throughout the annual cycle, circannual clocks affect methylation levels, stable methylation marks in the promoters of 30 candidate genes, embryo–adult transmission of methylation marks.

**Abstract:**

DNA methylation can change DNA properties, affecting chromatin accessibility, gene expression, and phenotypic variation. In clonal Norway spruce, warmer (WE) versus cooler (CE) embryogenic conditions produce phenotypically different trees. This climatic memory, induced during embryogenesis, remains stable in the resulting epitype trees, and the epigenetically altered timing of bud phenology persists between WE and CE epitypes even after decades under common garden conditions. We examined DNA methylation patterns in 14-year-old epitypes throughout the annual developmental cycle. Using targeted bisulfite sequencing, we screened for differential DNA methylation over a 3000 bp region in 2744 genes related to the epigenetic machinery, circadian clock, and phenology. Clustering DNA methylation differences in the CG context clearly separated epitype trees, confirming epigenetic mark differences. Differences in methylation of cytosines in all contexts were highly dynamic and varied markedly among annual developmental stages, suggesting the existence of circannual clocks affecting methylation levels in the studied genomic regions. Most stable methylation marks were identified in CG contexts, fewer in CHG and none in CHH contexts, consistent with differences in inheritance among methylation contexts. We identified stable CG and CHG methylation marks in the promoter regions of 30 specific genes. Two *ARGONAUTE* genes and 4 other genes exhibited stable marks across all time points for CE or WE, and putative embryo–adult transmission for some genes. These findings indicate that DNA methylation marks maintained in genomic regions throughout the annual cycle may contribute to an induced epigenetic memory established in embryos and later manifested as phenologically different epitype trees.

**Supplementary Information:**

The online version contains supplementary material available at 10.1007/s00425-026-05116-6.

## Introduction

The importance of epigenetic memory and other forms of plasticity in plant adaptation to climate change is an intriguing subject, particularly for species with very long generation time that have challenges to adapt quickly enough by classical Mendelian selection. Epigenetic modifications such as DNA methylation are important for chromatin structure, gene regulation, and in the repression of repetitive elements to retain genome stability (Zhang et al. 2018). The dynamic nature and importance of DNA methylation have also been implicated in biological phenomena, such as heterosis (Hamid et al. 2023), vernalization (Andrés and Coupland 2012), and stratification (Luján-Soto and Dinkova 2021), where the temporal aspects of epigenetic regulation play substantial roles. To what extent all the nuances of epigenetic regulation mechanisms are shared in the extant seed plants (angiosperms and gymnosperms separated by over 300 million years of evolution) is unclear, but DNA methylation changes have been implicated in epigenetic memory of stress and climatic impacts such as temperature change for both groups (Bräutigam et al. 2017; Fossdal et al. 2024). In the gymnosperm Norway spruce, contrasting temperature sums experienced during embryogenesis drive DNA methylation change differences, give rise to epitype trees with altered bud phenology, in a reproducible and predictable manner (Yakovlev et al. 2016; Carneros et al. 2017; Viejo et al. 2023). Genes related to the epigenetic machinery (DNA methyltransferases, *ARGONAUTE*) and *FTL* genes that control bud phenology are differentially methylated during somatic embryogenesis under these epitype-inducing (EpI) temperatures (Viejo et al. 2023), but it is unknown if these epigenetic mark differences at specific loci are mitotically maintained from the mature embryo through germination into the resulting Norway spruce trees and to what extent these marks are stably retained on the chromosomes through the annual growth cycle or not.

In classical quantitative genetics, phenotypic variation results from genotype and environmental variation as well as their interactions. However, epigenetics adds an extra dimension to this classical view of environment-caused variance (Hirsch et al. 2012; Diez et al. 2014). Epitypes, generated by somatic embryogenesis, giving genetically identical but phenotypically distinct individuals, are a very effective tool to study epigenetic modifications in a predictable and reproducible manner to potentially establish a causative link between specific epigenetic modifications and phenotypic behavior (Kvaalen and Johnsen 2008; Viejo et al. 2023; Fossdal et al. 2024) as long-lived species trees need to cope with multiple drastic and continuous changes of the environmental conditions. At the same time, their long generation times compared to most crops or herbaceous plants and the long juvenile phase of reproductive incompetence, up to several decades, are making the regular mechanisms of genomic adaptive selection not fast enough to ensure adaptation to environmental changes (Dauphin et al. 2021). Repetitive long-term climatic changes during evolution of tree species ensured the appearance and development of a genetically encoded machinery responsive for formation of epigenetic “memory” mechanisms (Biswas and Rao 2018; He and Li 2018). Thus, epigenetic mechanisms play a vital role in achieving a rapid enough adaptation to changing environments (Yakovlev et al. 2012; Bräutigam et al. 2013; Amaral et al. 2020; Kurpisz and Pawłowski 2022; Fossdal et al. 2024) through increased phenotypic variation by means of epigenetic memory.

Trees such as Norway spruce are perennial species with very long juvenile periods (breeding cycles usually > 15 years) and lifespan beyond 500 years (Castagneri et al. 2012). Trees pass through repeated annual cycles and respond consequently to seasonal changes, for example adapting the timing of bud phenology to avoid frost damage during bud burst in spring and bud and needle damage during winter. Fully acclimated buds and needles of boreal trees such as Norway spruce can survive immersion in liquid nitrogen (−196˚C) (Strimbeck et al. 2015), but do not tolerate frost when the buds flush and growth commence. Each annual cycle typically consists of de-hardening, bud flushing followed by growth during the spring, and bud set in summer in mature trees and in autumn in young seedlings, followed by a cold-hardened dormant period in winter to avoid frost damage until growth is restarted again by seasonal cues. The timing of these phenological events, as a result of climatic adaptation that synchronizes the plants’ annual growth-dormancy cycle with seasonal changes in temperature and photoperiod, is crucial for plant survival and competitive success (refs in (Olsen and Lee 2011; Singh et al. 2016). Trees are exposed to a gradient of temperature and photoperiod across their distribution range, leading many widespread species to seemingly exhibit heritable variation in the timing of phenology transitions as an adaptation to the local conditions, i.e., with continuous (clinal) variation (Howe et al. 2003; Olsen 2010). Due to their essential nature, bud phenology traits are believed to have high heritability and assumed to always be under strong classical genetic selection in trees (Howe et al. 2003). Despite the obvious importance of classical Mendelian inheritance, bud traits are also impacted epigenetically, exemplified by phenotypically distinct but genetically identical epitype trees. Temperature sum experienced during embryogenesis and seed development consistently and reproducibly induce an epigenetic memory that affect offspring phenology to a degree often ascribed to latitudinal and altitudinal ecotypes in Norway spruce (Yakovlev et al. 2012; Carneros et al. 2017), resulting in significant and long-lasting phenotypic changes in the progeny (Kvaalen and Johnsen 2008; Carneros et al. 2017; Fossdal et al. 2024). This epigenetic memory allows plants to exhibit priming effects, where prior exposure to stress conditions can lead to a more robust response in subsequent encounters (Duplan et al. 2025).

Epigenetic mechanisms involve DNA methylation, chromatin rearrangements, histone modifications, and non-coding RNAs in an interconnected fashion (Iwasaki and Paszkowski 2014). Stability of epigenetic modifications and heritability of epigenetic marks through mitosis or meiosis is the main prerequisite for their involvement in the formation and maintenance of epigenetic memory (Friedrich et al. 2018). DNA methylation is the most studied epigenetic mark in plants and has a role in normal development and mediate the transduction of external stimuli leading to changes in gene expression (Chinnusamy and Zhu 2009; Lucibelli et al. 2022). In the process of DNA methylation, methyl groups are covalently added to cytosine and adenine at the fifth position of the nucleoside in a double-stranded DNA, at the three different context sites CG, CHG, and CHH (H = A, T or C). DNA methylation modifies DNA molecule activity without changing its sequence. Cytosine DNA methylation has been shown to play a role in gene regulation (Zhang et al. 2018; Kumar and Mohapatra 2021), the silencing of transposable elements (TEs) (Slotkin and Martienssen 2007; Mirouze et al. 2009; Ramakrishnan et al. 2021), and chromosome interaction (Zhong et al. 2021; Lucibelli et al. 2022) in a tissue-specific manner, thus ensuring the proper growth, development, and function of an organism. At the phenotypic level, DNA methylation has been shown to affect the timing of developmental processes, such as bud dormancy, bud burst, flowering, and fertilization, and embryogenesis, thereby the reproduction process of trees and other plant species (Valledor et al. 2007; Viejo et al. 2012, 2023; Guevara et al. 2011; Basdeki and Hagidimitriou 2019; Klupczyńska and Ratajczak 2021).

The goal of this study was to establish if the epigenetic memory in clonal Norway spruce WE and CE epitype trees (Fig. S1) is associated with (i) stable DNA methylation mark differences during the entire annual cycle, (ii) dynamic patterns in these marks by calendar date or (iii) a combination of (i) and (ii). Conceptually, (i) and (ii) are indicators of two possible adjustment modes of the plant’s seasonal clock or annual rhythm-causing machinery. For that, we examined whether the extant epigenetic marks induced during the somatic embryogenesis under epitype-inducing temperature conditions (Viejo et al. 2023) are maintained in the resulting epitype trees. Due to the massive size of the Norway spruce genome (about 20 Gbp; Nystedt et al. 2013), we used a targeted approach to address the DNA methylation status at a single-base resolution in specific target gene regions of 2744 selected genes involved in the epigenetic machinery and adaptive trait formation. To this end, detailed bud phenology was recorded to verify the epigenetic shift in bud phenology in the epitypes and DNA methylation patterns in apical buds, and shoot tips were characterized at 10 calendar dates spread throughout the year for the CE and WE epitypes.

## Materials and methods

### Plant material, phenological observations, and sample collection

In this study, we used clonal epitype trees of Norway spruce (*Picea abies* (L.) Karst.) of the B10V clone, produced by somatic embryogenesis from one zygotic embryo (genotype15884) under 18 °C (cool embryogenesis giving rise to the cool epitype, CE) or 28 °C (warm embryogenesis giving rise to the warm epitype, WE). The seed (with the zygotic embryo) of the B10V genotype originated from a controlled cross of grafted *Picea abies* (L.) Karst. parents (♀#2650 × ♂#2707) in greenhouse conditions in 1998 (Kvaalen and Johnsen 2008). The conditions during the somatic embryogenesis and cultivation of the epitype embryos and seedlings/young plants were as previously described (Johnsen et al. 2005a, b; Kvaalen and Johnsen 2008; Yakovlev et al. 2014). Two-year-old epitype seedlings were planted in 2007 in a unique common garden epitype trial of Norway spruce in Hogsmark (Ås, Norway; 59° 40′ 07″ N 10° 43′ 04″ E). The trees were shown to have a stable epigenetic memory of temperature during the somatic embryogenesis (Kvaalen and Johnsen 2008; Carneros et al. 2016).

Detailed phenological observations for the CE and WE epitype trees during spring bud burst. For phenological observations, eight 11-year-old trees of the B10V clone from each of the CE (trees 3677, 3668, 3669) and WE (trees 3662, 3667, 3678) epitype trees of the B10V clone were used and the subsequent elongation growth and bud set were performed using a discrete phenological scale coded from 0 till 8 (Krutzsch 1973) once a week during 8 consecutive weeks from April 27 until June 16 in 2016. Data from the individual trees of each epitype were recorded for the most apical bud/shoot tip and selected apical buds/shoots in branches from the four previous growth years. Mann–Whitney tests were used to assess the shift in bud burst-timing between the two epitypes.

**Fig. 1:**
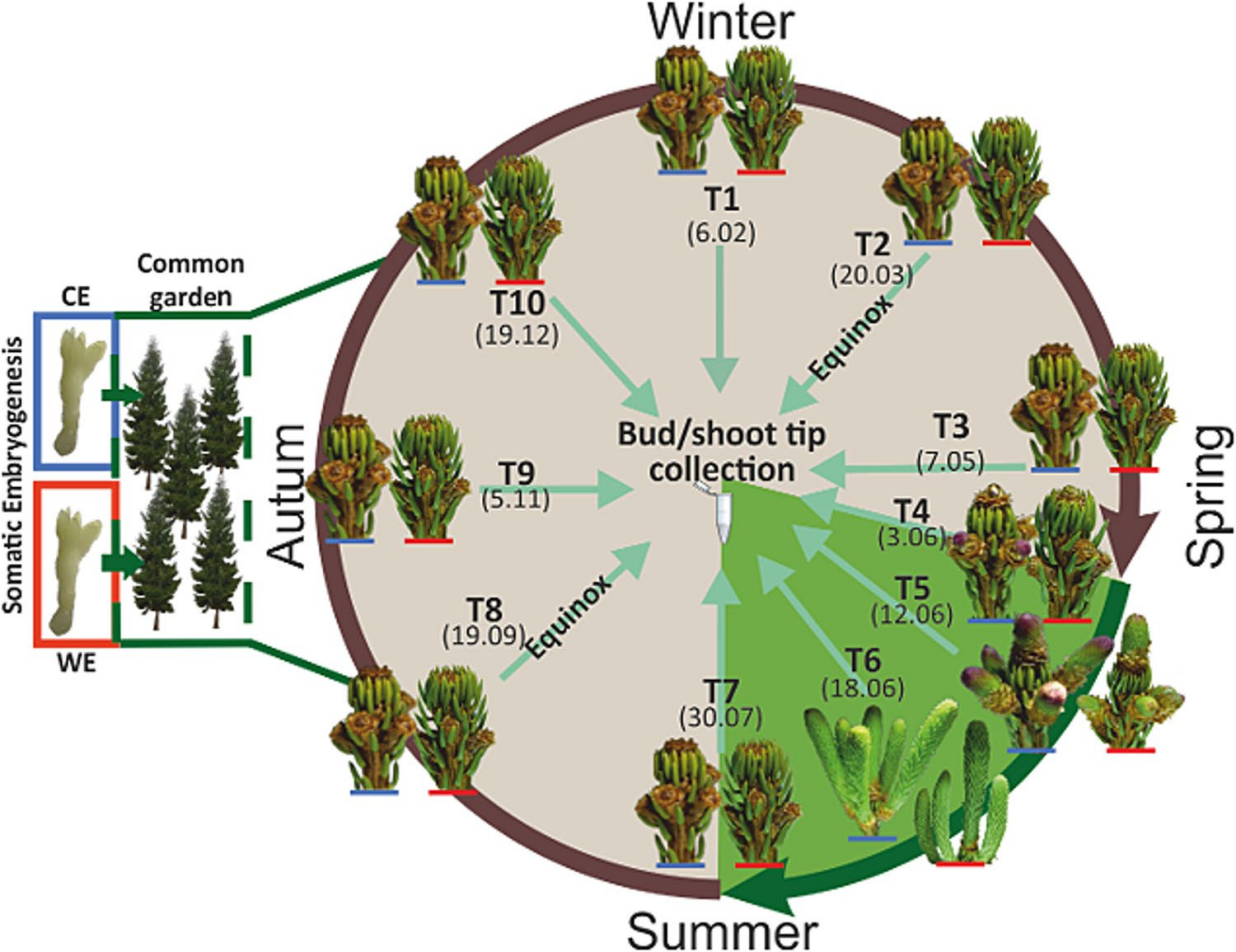
Annual cycle of development of Norway spruce epitype trees, indicating the 10 time points (T1–T10, dates in parentheses) when buds or apical shoot tips of clonal epitype trees (clone B10V) were collected. Individuals originated from somatic epitype embryos developed at 18 °C (CE, in blue) or 28 °C (WE, in red) that gave rise to 14 years old trees, growing under common garden conditions

For analysis of DNA methylation, shoot tips/terminal buds of 14-year-old trees were collected in 2019 from the south side of the first and second whorl of branches from six trees of the two epitypes (CE trees: 3677, 3668, 3669; WE trees: 3662, 3667, 3678) at ten time points throughout the annual cycle; T1–06.02, T2–20.03, T3–07.05, T4–03.06, T5–12.06, T6–18.06, T7–30.07, T8–19.09, T9–05.11 and T10–19.12 (Fig. 1). The samples were flash-frozen in liquid nitrogen until further processing. Collection dates were selected to cover the annual cycle with relatively fixed intervals with 3 additional time points in June to capture the differences in bud development status and subsequent DNA methylation patterns.

### DNA extraction

Total DNA was extracted from the 60 samples. A powdered tissue of several shoot tips/terminal buds (buds) without expanded needles was ground in liquid nitrogen with a mortar and pestle. The total DNA was extracted using the DNeasy Plant Mini Kit (Qiagen, Hilden, Germany, cat. no. 69104) according to the manufacturer’s instructions. The total DNA preparations were stored at − 80 °C. The concentration and quality of the DNA were tested with a Nanodrop 2000 Spectrophotometer (Thermo Fisher Scientific, Waltham, MA, USA). Additionally, the integrity and quantity of total DNA were assessed by an Agilent 2100 Bioanalyzer with an Agilent DNA 7500 Kit (Agilent, Santa Clara, CA, USA # 5067–1506).

### Targeted bisulfite sequencing and estimation of DNA methylation

Targeted bisulfite sequencing (BS) and data processing was performed as described in Viejo et al. (2023). Briefly, the samples were fragmented using an M220 Focused-ultrasonicator (Covaris Inc., Woburn, MA, US), the DNA fragments were bisulfite-converted using the EZ DNA Methylation-Gold Kit (Zymo Research Corp, Orange, CA, USA), and the libraries were made using the SeqCap Epi Enrichment system (Roche, Basel, Switzerland), following the manufacturer´s instructions (Wendt et al. 2018). For analysis, we targeted a total of 2744 gene regions of 3 kb length (2 kb of the promoter and 1 kb of the gene body at either side of the transcriptional start site (TSS) with a focus on genes involved in the timing of phenology, the entire epigenetic machinery, and the circadian clock. The probe design is available from Roche (OID42175). Additional information about the targeted BS sequencing and the list of the targeted genes is described in Viejo et al. (2023).

Pooled captured bisulfite-converted DNA libraries were sequenced at the BGI Genomics (Hong Kong, China) using a DNBSEQ (G-400) sequencing platform with 150PE with 40% in lane control sequencing. Using version 1 of the Norway spruce genome (Nystedt et. al. 2013), the BS sequencing target sequences were expanded with 500 base pairs in both directions. This was done to allow for efficient aligning of flanking reads. Paired-end reads were aligned and base-resolution DNA methylation levels extracted using the software Bismark, v0.22.3 (Krueger and Andrews 2011) with the coverage2cytosine module. Hierarchical clustering of samples was done using the R package methyl kit (version 1.26.0) (Akalin et al. 2012). The distance measure used was’correlation’, and’ward’ was used as the agglomeration method.

Differentially methylated target sequence regions were identified using the R package DMRcaller (version 1.32.0) (Catoni et al. 2018). All contexts were analyzed (CG, CHG and CHH), and a sliding window-based approach was used. The minimum size of the window was set to 50 base pairs, containing a minimum of 4 cytosine positions. Proportional difference in methylation levels was required to be at least 0.1 for a position to be included and a *P*-value cutoff of 0.01 was used.

## Results

### Earlier bud burst in the CE than the WE epitype trees

**Fig. 2:**
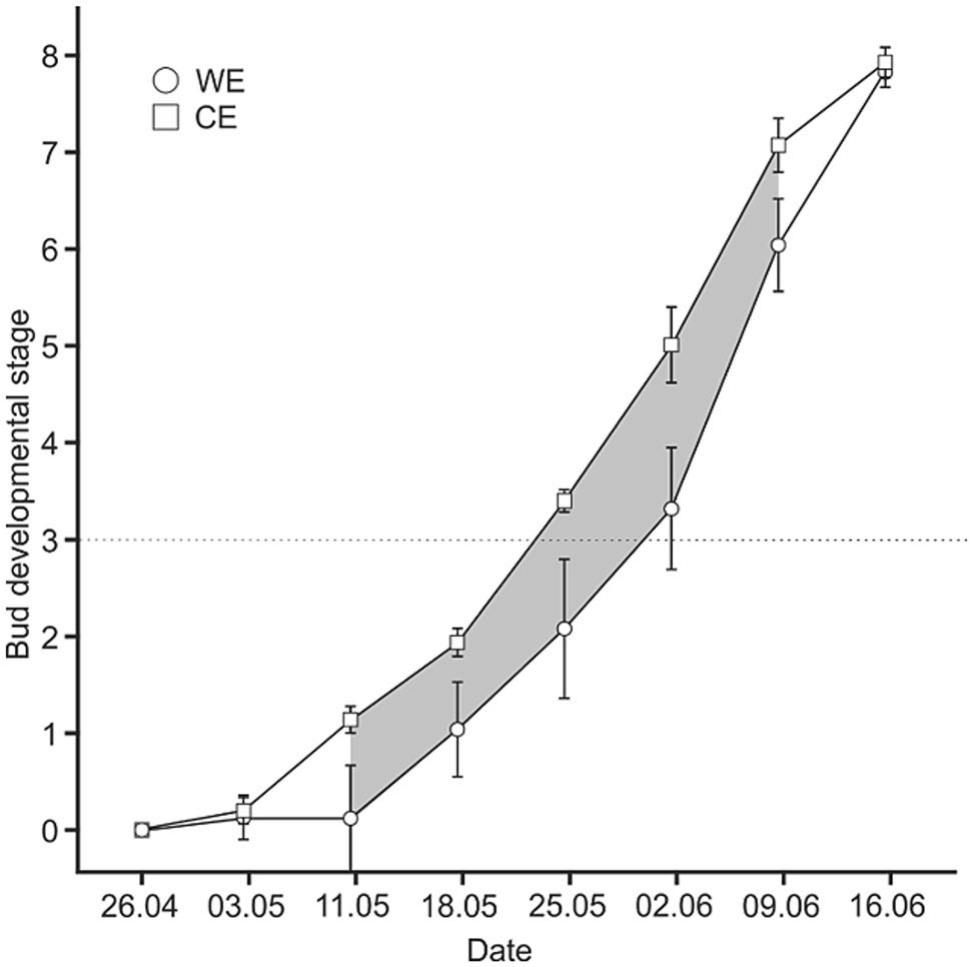
Timing of bud development in 11-year-old clonal WE and CE epitype trees of Norway spruce (clone B10V, after 9 years under common garden conditions). The gray area delineates the time periods with statistically significant differences in bud development between epitypes. Bud stages follow Krutzsch’s scale: 0= dormant buds 1 = buds slightly swollen, needles bent backwards and outwards 2 = buds swollen, green to gray-green in color, bud scales still closed, 3 = burst of bud scales, tips of needles emerging (indicated as a dotted line in the figure), 4 = first elongation of needles to about double bud length, 5 = first spread of needles, buds have the appearance of a painter’s brush, 6 = elongation of the shoot, basal needles not yet spread, 7 = differentiation of shoot, basal needles spread, 8 = all needles more or less spread, new buds developing. Results are mean values from 3 individual trees of CE and 5 individual trees of WE with recordings for the shoot apex and the 4 uppermost whorls of branches. Significance was calculated with Mann–Whitney test and 95% confidence intervals are represented

To verify that CE and WE epitype trees retain the embryogenesis-induced epigenetic memory after more than a decade of common garden cultivation, we first conducted detailed observations of their spring bud burst progression. The differences in bud burst phenology in the WE and CE epitypes were clearly visible in the stand (Suppl. Fig. S1). Detailed recordings of bud burst phenology showed that bud swelling in CE trees occurred on average during the week of May 3, whereas in WE trees it occurred during the week of May 11; actual bud burst occurred during the weeks of May 18 and May 25, respectively (Fig. 2). Statistical analysis revealed significant differences in bud burst progression between the two epitypes from May 11 to June 9 (Fig. 2). These results confirm that the temperature-induced epigenetic memory during embryogenesis remains stably expressed under field conditions after more than a decade, establishing a reliable biological premise for subsequent investigation of the DNA methylation basis of this memory.

### Overall DNA methylation in the targeted genomic regions differs between the clonal CE and WE epitype trees

**Fig. 3:**
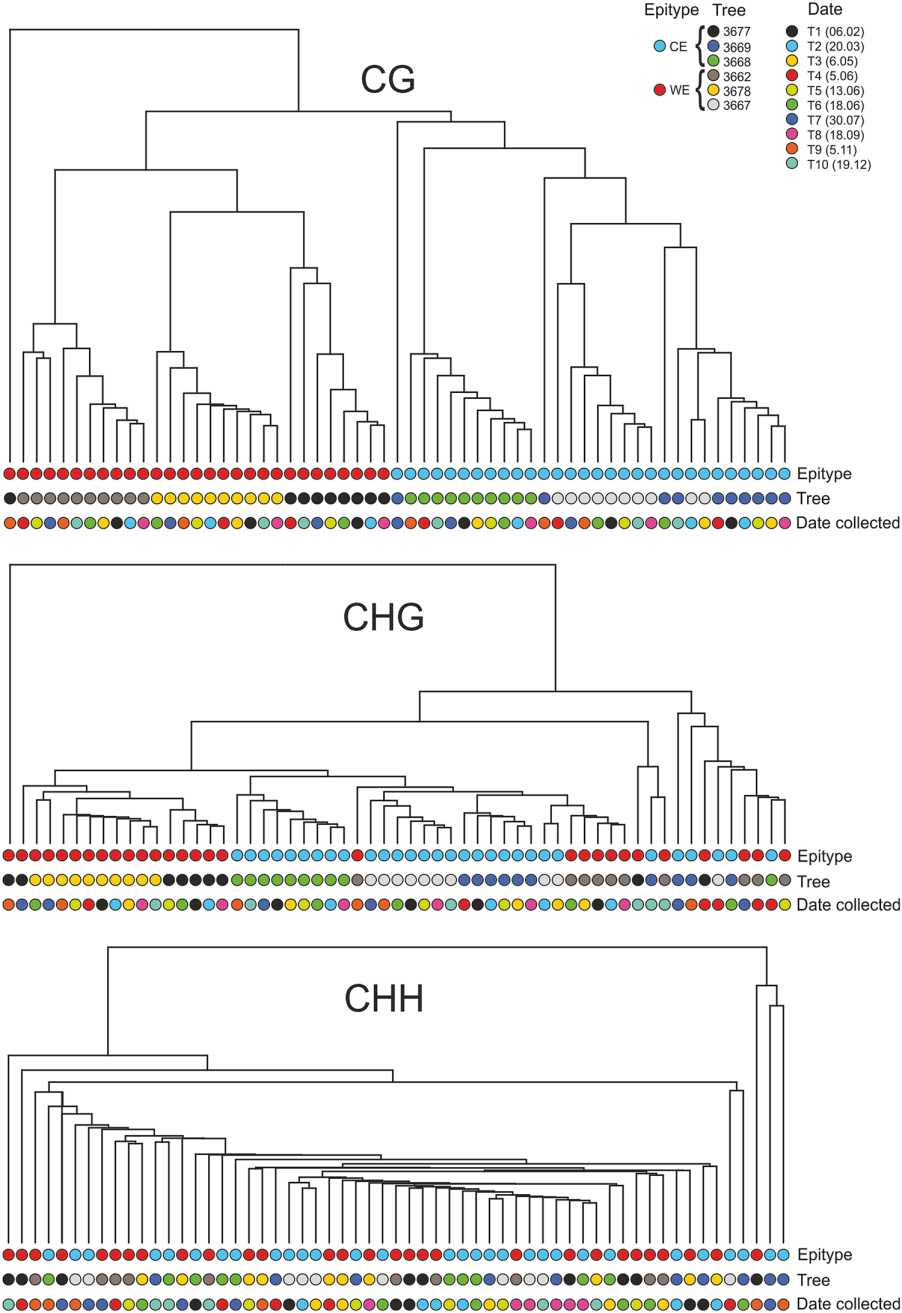
Clustering of the cytosine methylation in all three DNA methylation contexts (CHH, CHG (H = A, T or C) and CG) in 2 kb of the promoter and 1 kb of the gene body at either side of the transcriptional start site of 2744 targeted genes in clonal epitype trees originating from somatic embryos developed under 18 °C (CE; blue) or 28 °C (WE; red) of the clone B10V. Bud/shoot tip samples from 10 time points (T1–T10, dates in parentheses) throughout the annual cycle were analyzed for 3 individual trees of each epitype at each time point

To investigate the molecular basis of epigenetic memory in CE and WE epitype trees, we first performed cluster analysis of the overall methylation patterns of all methylated cytosines within the targeted 3 kb regions of 2,744 selected genes. Cluster analysis of the samples (corresponding to individual trees) based on the total number and distribution of methylated cytosines in the targeted 3 kb region of the 2744 selected genes revealed marked differences between the two epitypes for the CG methylation context (Fig. 3). In this context, the epitypes and individual trees within each epitype are grouped clearly according to their methylation patterns (Fig. 3). Six distinguished clusters were detected, corresponding to individual trees, which were further combined into two main clusters representing the two epitypes. Only 6 out of 30 CE samples and one out of 30 WE samples showed mixing between clusters representing individual trees. In the other methylation contexts (CHG and CHH), the clustering was less clear. In the CHG context, two large clusters were observed, partially corresponding to the epitypes. However, 18 samples either shifted cluster affiliation or formed outlier clusters. In the CHH context, no structure in sample distribution between the epitypes was detected. This result demonstrates that DNA methylation patterns in the CG context exhibit stable epitype specificity and can serve as molecular markers for distinguishing the two epitypes.

The distribution of methylated cytosines along the analyzed region of the 2744 selected genes showed clear patterns across all contexts. The analyzed promoter region (first 2 kb) exhibited several times higher DNA methylation level than the analyzed first 1 kb of the coding region but exhibited a progressive reduction toward the TSS. This coding region had a consistently low methylation level (Suppl. Fig. S2).

### Seasonal variation in DNA methylation patterns

**Fig. 4:**
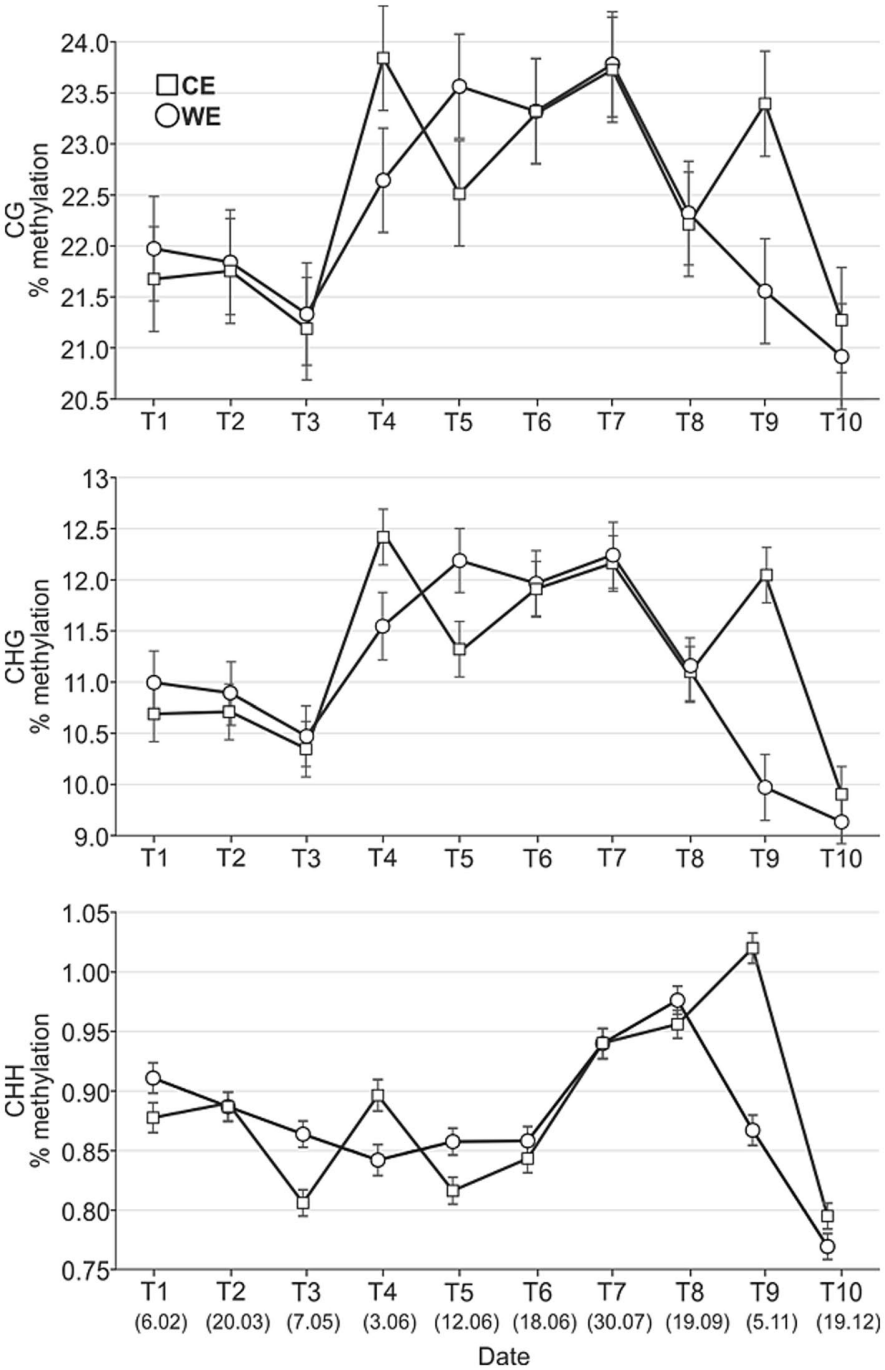
Overall seasonal levels of DNA methylation for the CG, CHG and CHH contexts in 3 kb regions of 2744 targeted genes in clonal Norway spruce CE and WE epitype trees (clone B10V). Percentage was calculated as the number of methylated cytosines (Cme) divided by the number of total C. Bud/shoot tip samples were collected at 10 time points (T1–T10; dates in parentheses) throughout the annual cycle from 3 individual trees of each epitype. Standard deviation is shown

Analysis of DNA methylation in the targeted region of the 2744 genes at the ten time points throughout the annual cycle in the CE and WE revealed that the overall (average) methylation levels varied by context and season. DNA methylation was the highest in the CG (21–24%) and CHG (10–13%) contexts, while the CHH context showed only around 1% methylation (Fig. 4). Seasonal variation in methylation level was evident for CG and CHG, with increased methylation during the growth period (June 3 to July 30) compared to dormant phases. Except for CHH, where a higher methylation level was during early dormancy (September–December). In all contexts, significant differences between epitypes were also observed: CE exhibited higher methylation than WE at T4 (June 3) and T9 (November 5), whereas WE showed higher levels than CE at T5 (June 12) (Fig. 4). Elevated methylation at T4 and T5 coincided with active shoot elongation (from early to mid-June) with a time shift between epitypes. There was a pronounced drop in methylation in WE compared to CE during November (T9), possibly reflecting differential processes during winter acclimation. Despite the CHH methylation remaining very low, the trend of CHH methylation differences between epitypes at T4 and T9 is identical to that of the CG and CHG contexts.

### Differential methylation patterns between epitypes throughout the annual cycle

**Fig. 5:**
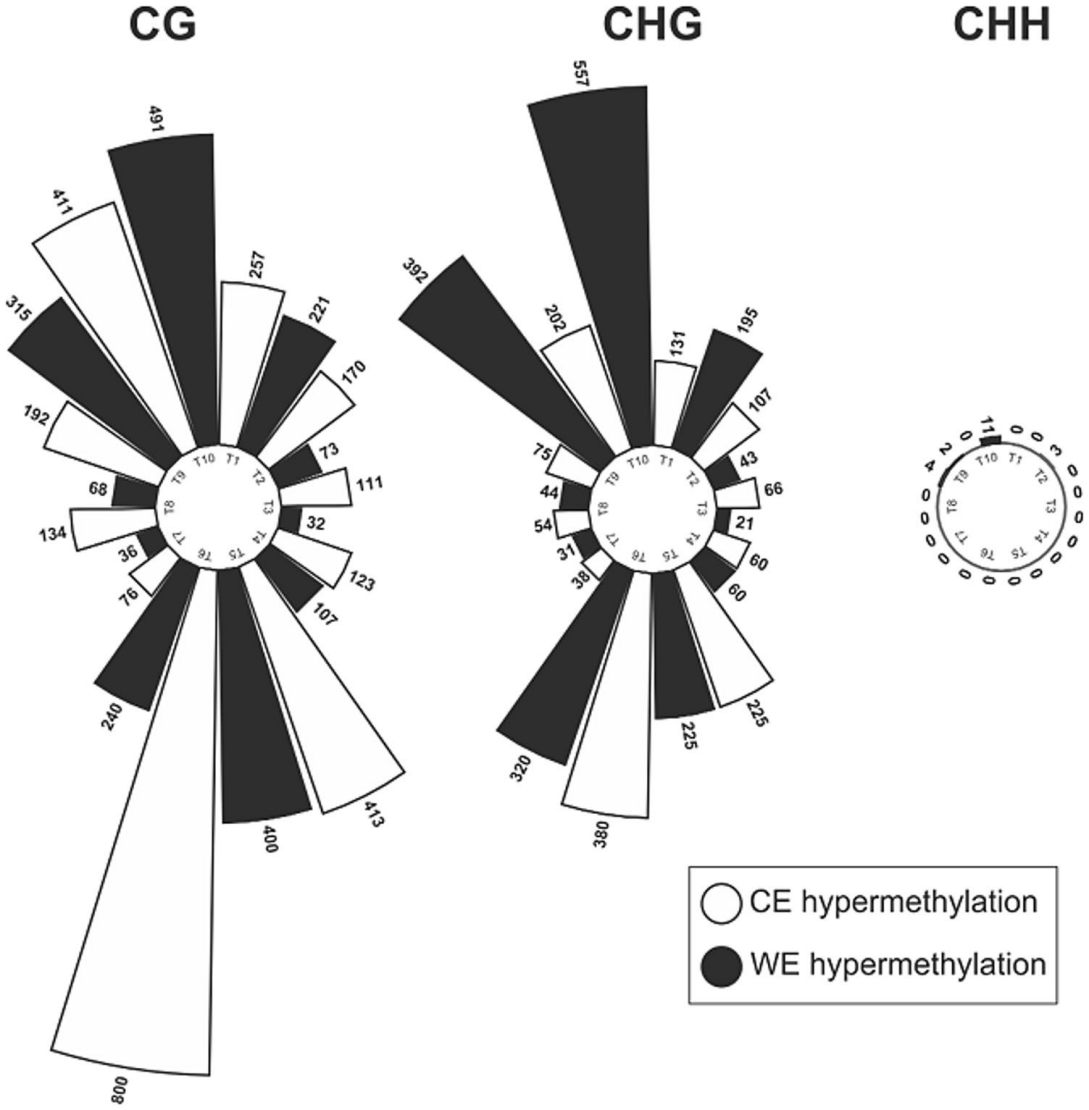
Polar area diagram displaying the number of differentially methylated regions (DMRs) for CG, CHG and CHH methylation contexts in buds/shoot tips of clonal Norway spruce WE and CE epitype trees (clone B10V) at 10 time points (T1–T10, dates in Fig. 2) throughout the annual cycle. Three individual trees of each epitype were analyzed per time point

The highest number of differentially methylated regions (DMRs) (both hyper- and hypo-methylated) between the CE and WE epitypes for the CG and CHG contexts was found during two periods: T8–T1 (from September 19 to February 6) and T5–T6 (June 12–18) (Fig. 5). In the CHH context, significant DNA methylation differences were found for T9–T10 only (from November 5 to December 19; 6 and 11 DMRs). In total, there were 3756 DMRs for all three contexts during autumn and winter periods (T8–T1). Of these, there were 2089 DMRs for CG, 1650 DMRs for CHG, and 17 DMRs for CHH. In June (T5–T6), there were 3003 DMRs in total with 1853 DMRs for CG and 1150 DMRs for CG.

**Fig. 6:**
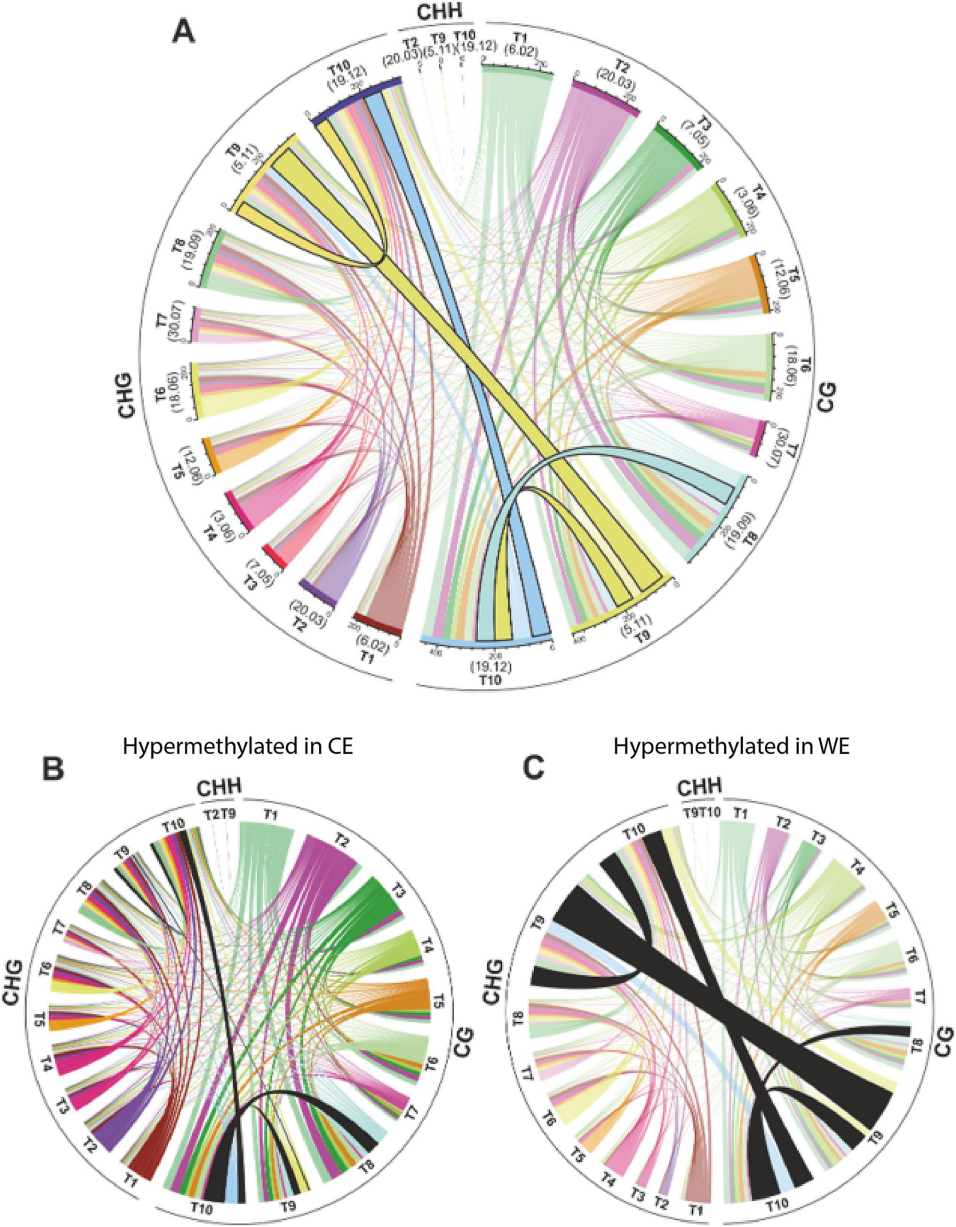
Sharing of differentially methylated genes (DMGs) for CG, CHG, and CHH methylation contexts between 10 time points throughout the annual cycle for buds/shoot tips of clonal Norway spruce CE or WE epitype trees (clone B10V). Black lanes highlight the strongest correspondences between contexts. **A** DMGs between the two epitypes shared among time points (T1–T10; dates in parentheses). **B** Hypermethylated in CE: share of DMGs with increased DNA methylation present in the CE compared to WE among time points. **C** Hypermethylated in WE: shared DMGs fragments with increased DNA methylation in the WE compared to CE among time points. Three individual trees of each epitype were analyzed per time point

Comparing differentially methylated genes (DMGs) between epitypes that were shared between different time points, the highest number of common DMGs was found during autumn and winter periods at T8–T10 (from September 19 to December 19) (Fig. 6A). As expected, connections among DMGs were observed between the CHG and CG contexts. DMGs with increased methylation in the CE versus WE were predominantly shared between T8 (September 19) and T10 (December 19) and T9 (November 5) and T10 for the CG context and between T9 and T10 for CHG (Fig. 6B). Similarly, the decreased number of DMGs for the CE was mostly shared between T8 and T10, and T9 and T10 for the CG context and T9 and T10 for CHG context (Fig. 6C).

### Persistent differential methylation of multiple genes between epitypes throughout the annual cycle

**Fig. 7:**
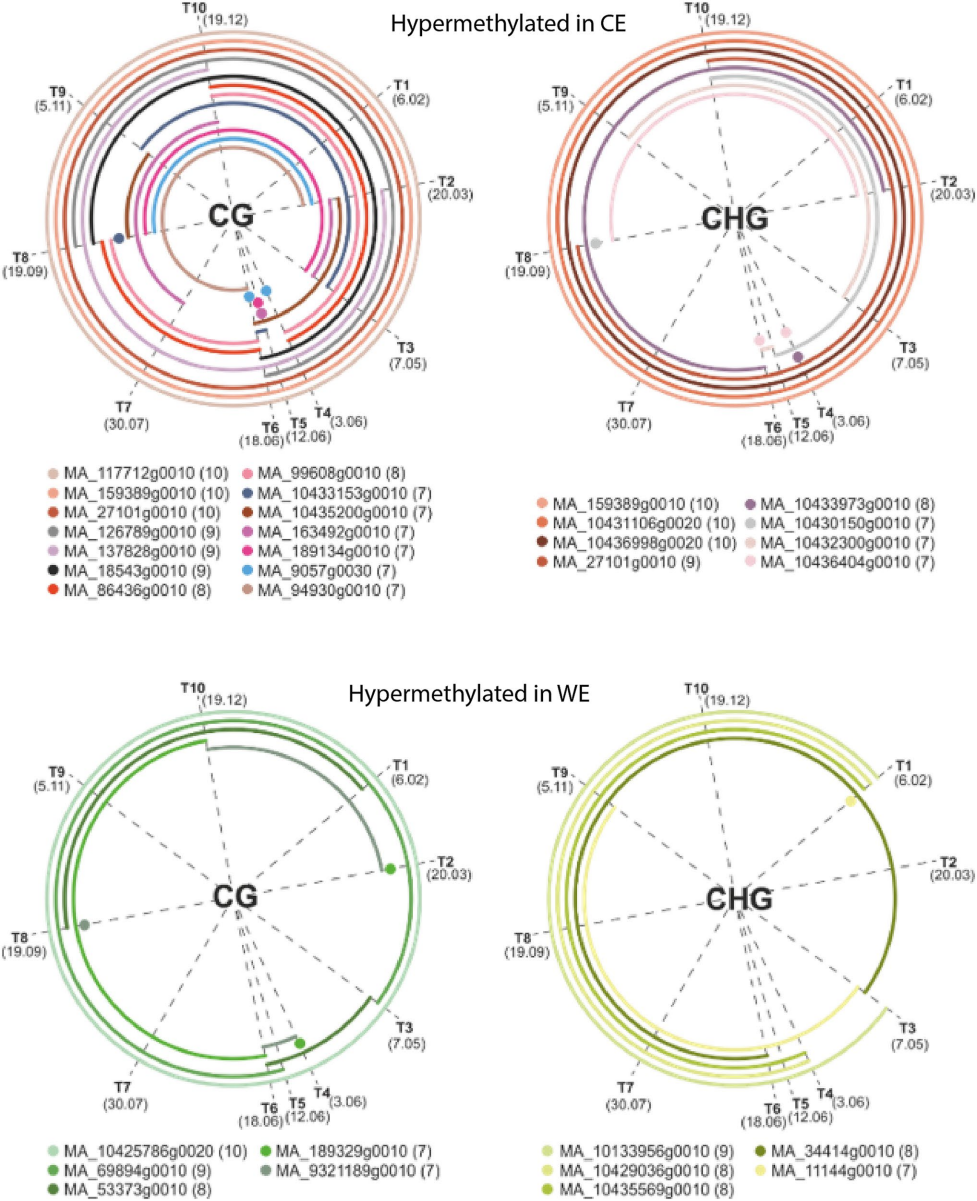
Stability of differentially methylation genes (DMGs) in buds/shoot tips at different time points (T1–T10, dates in parentheses) throughout the annual cycle between clonal Norway spruce CE and WE epitype trees. The graph represents DMGs of CG and CHG contexts that were hyper-/hypo-methylated in at least three consecutive time points. Numbers in parenthesis after gene models indicate the number of time points at which there were methylation differences between epitypes. Three individual trees of each epitype were analyzed per time point

Within the targeted 3000 bp region of the 2744 genes analyzed, a total of 1548 DMRs between the epitypes were identified, each occurring at least once during the annual cycle. These DMRs were distributed across 479 DMGs (Suppl. Table S1.2), of which 30 exhibited consistent differential methylation (either hyper- or hypo-methylation) in seven or more time points throughout the annual cycle (Fig. 7; Table 1; Suppl. Table S1.1.). Of these, 30 DMGs 19 showed differential methylation in the CG context and 13 genes in CHG. Overall, the most persistent DMRs were observed during dormant and quiescent autumn-to-spring periods (T8–T2; from September to March).

**Table 1:** List of the stable differentially methylated genes (DMGs) that were hyper-/hypo-methylated in at least seven time points throughout the annual cycle of development in the shoot tips/buds of clonal Norway spruce CE and WE epitype trees

	Gene ID	Gene name and annotation based on arabidopsis or poplar orthologs	C^me^ context	Number of time points	Methyl. pattern in CE
1	MA_159389g0010	*PaAGO2* – ARGONAUTE2	CG, CHG	10, 10	gain
2	MA_27101g0010	*PaCRK42* – CYSTEINE-RICH RLK (RECEPTOR-LIKE) PROTEIN KINASE 42	CG, CHG	10, 9	gain
3	MA_10425786g0020	*PaAGO7* – ARGONAUTE7	CG	10	loss
4	MA_117712g0010	*PaAP1* – Eukaryotic aspartyl protease family protein	CG	10	gain
5	MA_10431106g0020	*PaTGH*, TOUGH – RNA-binding and -processing proteins	CHG	10	gain
6	MA_10436998g0020	*PaKMT2* – Histone–Lysine *N*-Methyltransferase 2, SET domain protein	CHG	10	gain
7	MA_69894g0010	*PaUNE2* – UNFERTILIZED EMBRYO SAC 2; Major facilitator superfamily protein	CG	9	loss
8	MA_126789g0010	*PaBEBT1* – Benzyl alcohol O-benzoyltransferase (BEBT); HXXXD-type acyl-transferase family protein	CG	9	gain
9	MA_137828g0010	*PaERD13* – Early dehydration-induced 13, ERD13/glutathione *S*-transferase PHI10	CG	9	gain
10	MA_18543g0010	*PaCPK17* – Calcium-Dependent Protein Kinase 17, CPK17	CG	9	gain
11	MA_10133956g0010	*PaGID1* – GA-insensitive—DWARF1C, GID1C	CHG	9	loss
12	MA_53373g0010	*PaMYB109* – MYB family TF	CG	8	loss
13	MA_86436g0010	*PaDUF247* – transmembrane protein, putative (DUF247)	CG	8	gain
14	MA_99608g0010	*PaGER5* – GEM-related 5;GER5	CG	8	gain
15	MA_10429036g0010	*PaTERT* – Telomerase Reverse Transcriptase, TERT	CHG	8	loss
16	MA_10435569g0010	*PaUBC23* – Ubiquitin-Conjugating Enzyme 23	CHG	8	loss
17	MA_34414g0010	*PaHIPP41* – Heavy metal-associated isoprenylated plant protein 41;HIPP41	CHG	8	loss
18	MA_10433973g0010	*PaCYS6* – PHYTOCYSTATIN 6	CHG	8	gain
19	MA_189329g0010	*PaTBL36* – TRICHOME BIREFRINGENCE-LIKE gene family	CG	7	loss
20	MA_9321189g0010	*PaDRP1* – probable disease resistance protein DRP	CG	7	loss
21	MA_10433153g0010	*PaTPR1* – Penta-/Tetra-tricopeptide repeat (TPR)-like superfamily protein	CG	7	gain
22	MA_10435200g0010	*PaSPL2* – Putative RING-type E3 Ubiquitin ligase family protein, SPL2	CG	7	gain
23	MA_163492g0010	*PaPOLA1a* – DNA-directed RNA polymerase subunit alpha	CG	7	gain
24	MA_189134g0010	*PaJRL* – Jacalin-related lectin	CG	7	gain
25	MA_9057g0030	*PaGRF1* – Growth-Regulating Factor, zinc finger/Zinc knuckle protein	CG	7	gain
26	MA_94930g0010	*PaPB1* – Octicosapeptide/Phox/Bem1p family protein	CG	7	gain
27	MA_11144g0010	*PaCER1* – ECERIFERUM 1-like, Fatty acid hydroxylase superfamily	CHG	7	loss
28	MA_10430150g0010	*PaDRH1* – pre-mRNA-splicing factor ATP-dependent RNA helicase DEAH4, RNA helicase family protein	CHG	7	gain
29	MA_10432300g0010	*PaUaP1* – Uric acid–xanthine permease protein	CHG	7	gain
30	MA_10436404g0010	*PaRDL1* – Rhodanese-like/Cell cycle control phosphatase superfamily protein	CHG	7	gain

**Fig. 8:**
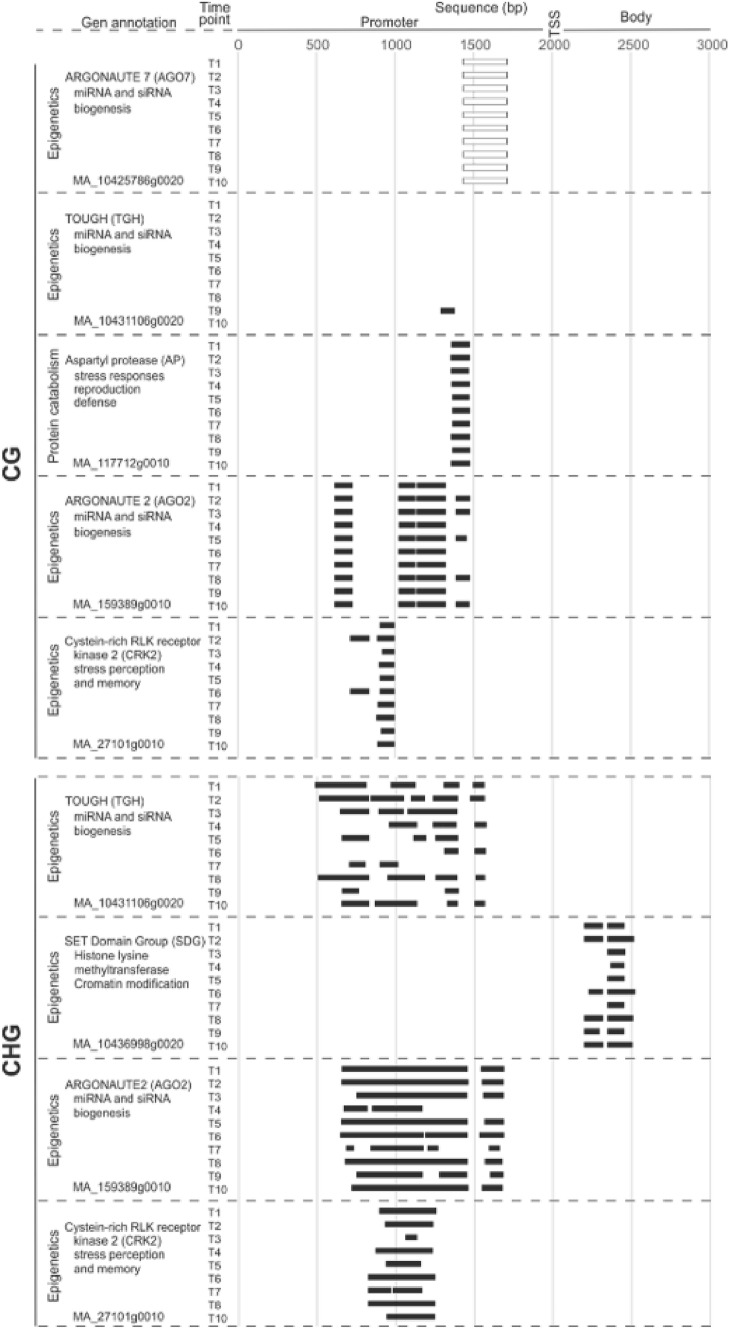
Distribution of differentially methylated regions (DMRs) in CG and CHG contexts within 3 kb of stably differentially methylated genes at all time points (T1–T10, dates in Fig. 2) throughout the year in buds/shoot tips of clonal Norway spruce CE and WE epitype trees. Transcription start site (TSS) is at 2000 bp. Empty bars, hypermethylated DMRs in WE; dark bars, hypermethylated in CE. Three individual trees of each epitype were analyzed per time point

Six DMGs exhibited differential methylation between epitypes across all sampling time points throughout the annual cycle (Table 1, Fig. 7). The distribution of DMRs between epitypes within these DMGs across the ten sampling points during the annual cycle is shown in Suppl. Table S2. Two of these genes (MA_159389g0010 (*ARGONAUT 2* ortholog – *PaAGO2*) and MA_27101g0010 (*CYSTEIN-RICH RECEPTOR-LIKE PROTEIN KINASE 42 – PaCRK42*), were hyper-methylated in both contexts in the CE epitype (Fig. 7). Another gene encoding an Argonaut-like protein MA_10425786g0020 (*PaAGO7*) was hyper-methylated throughout the year in the CG context in WE. For *PaAGO2*, the greatest DNA methylation differences between epitypes occurred during T9–T3 (from November 5 to May 7) and T6–T7 (from June 18 to July 30), whereas *PaAGO7* showed the largest difference at T2 (March 20). The other three genes that were differentially methylated throughout the year included MA_10431106g0020 (*PaTGH1a*, ortholog of *TOUGH* protein), MA_10436998g0020 (*PaKMT2*, SET domain protein) and MA_117712g0010 (*PaAP1*, Eukaryotic aspartyl protease family protein). Five out of six stable DMGs were hypermethylated for the CE while only one, MA_10425786g0020 (*PaAGO7*), was hypermethylated in WE (Fig. 8, Table 1). These genes were stably differentially methylated between epitypes in any of CHG and CG contexts. Considering probable functions of these DMGs, three of them could be involved in miRNA pathways (*PaAGO2, PaAGO7, PaTGH1a)*, and the other three could be involved in histone methylation (*PaKMT2*) and defense and stress response (*PaAP1, PaCRK42*).

**Fig. 9:**
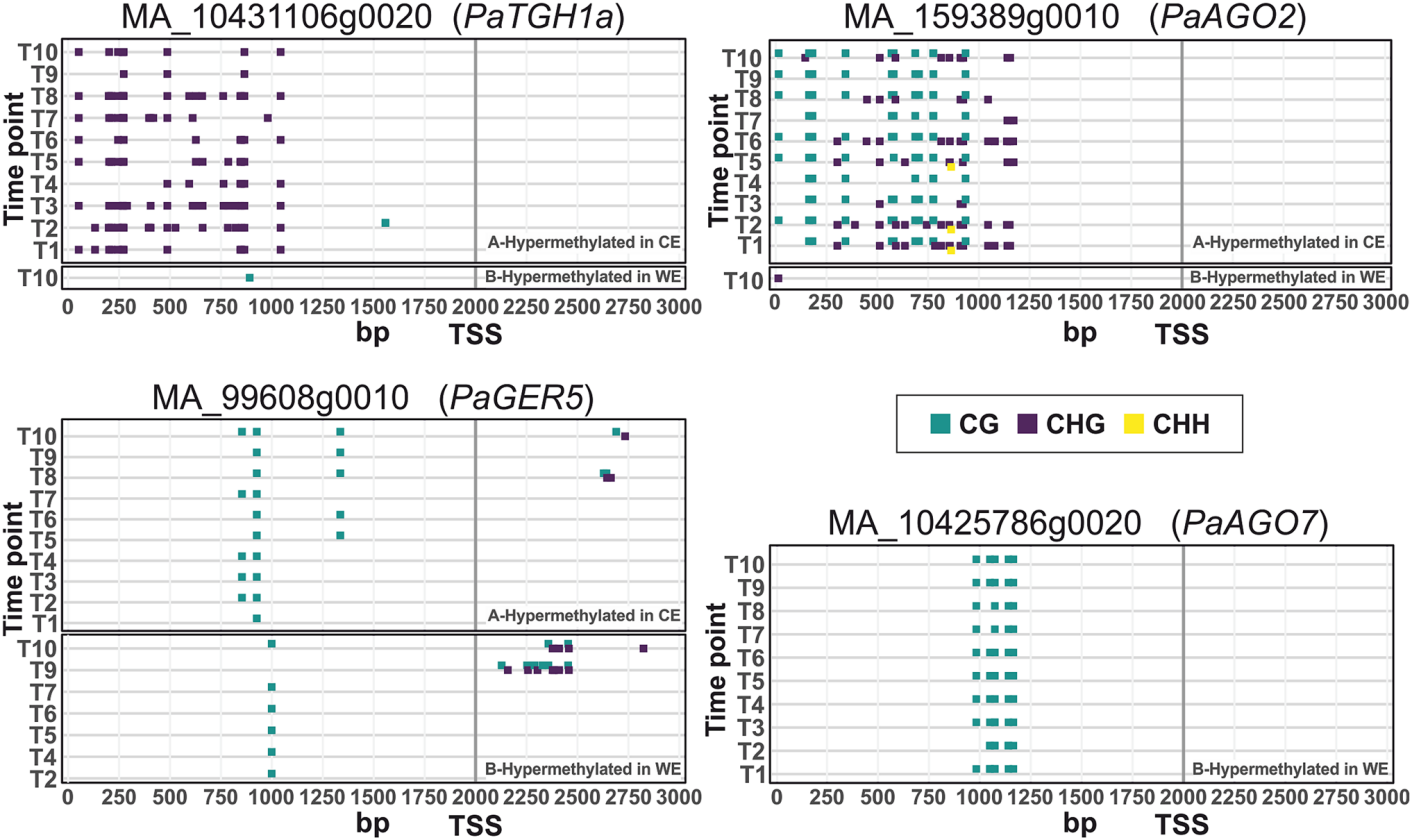
Specific locations (positions) of the differentially methylated sites (DMS) where the addition (**A**) or removal (**B**) of a methyl group in CE significantly varies between epitypes for the selected genes. *PaTGH1a*, TOUGH—RNA binding and processing proteins. It is an important component of miRNA and siRNA biogenesis. *PaGER5*, GRAM domain family protein, encodes a protein with unknown function that could be involved in hormone mediated regulation of seed germination/dormancy. *PaAGO2* and *PaAGO7*, ARGONAUTE-like proteins, act as the catalytic engine of the RNA-induced silencing complex (RISC). More examples of DMS locations in selected genes presented in the Suppl. Fig. S4

In addition to DMRs, we tried to analyze the specific locations of the differentially methylated sites (DMS) where the addition or removal of a methyl group on the cytosines in different contexts significantly varies between epitypes (Suppl. Table S3). Due to the extremely dynamic nature of DNA methylation at the single cytosines level, we are presenting here only a few examples of DMSs distribution along the studies region of selected genes (Fig. 9 and Suppl. Fig. S4). For all analyzed genes, stable DMSs were placed in the promoter part of the fragments. For two differentially methylated Argonaute genes, the patterns and distribution of differentially methylated cytosines were completely different. For *PaAGO7*, there were seven hypermethylated cytosines of CG context only in each time point for WE in the only region 983–1165 bp from the start of the 3 kb fragment. There were also variations in methylation of specific cytosines, e.g., no methylation at position 983 in T2, but methylation in position 1140 instead, absent in all other time points. But for *PaAGO2*, the methylation pattern looked to be more complicated. It contained DMSs hypermethylated in CE of all three contexts, but only some cytosines in CG context were stably hypermethylated in all time points. For *PaGER5*, some cytosines were hypermethylated for both CE and WE in the CG and CHG contexts, but only cytosines at positions 926 were stably hypermethylated in CE at all time points. For *PaTGH1a*, most of cytosines in the CHG context were hypermethylated in CE in all time points with some variation in specific positions. Hence, on single-base resolution level, we got very precise methylation patterns of specific cytosines for each gene. It corresponds in general to a more generalized DMRs analysis. Hence, both approaches were used for analyzing methylation patterns dynamics between epitypes. Comparisons of DNA methylation patterns were studied between somatic embryos and adult trees of CE and WE.

To assess whether temperature-induced DMRs during somatic embryogenesis are mitotically transmitted to buds in the resulting trees, suggesting an involvement in the epigenetic memory, we compared the DNA methylation in shoot tips/buds of the CE and WE epitypes with previously published data on the same 2744 genes in the apical part of corresponding CE and WE embryos (shoot apical meristem (SAM)-containing part; Viejo et al. 2023) of the same genotype, B10V. In both the epitype trees and embryos, the DNA methylation was the highest in the analyzed 2 kb promoter region and decreased toward the TSS with lower levels in the subsequent 1 kb coding region (Suppl. Fig. S3). In the CHG and CG contexts, the overall methylation level was relatively similar between the epitype trees and the SAMs of embryos, whereas CHH methylation was markedly lower in the trees.

**Fig. 10:**
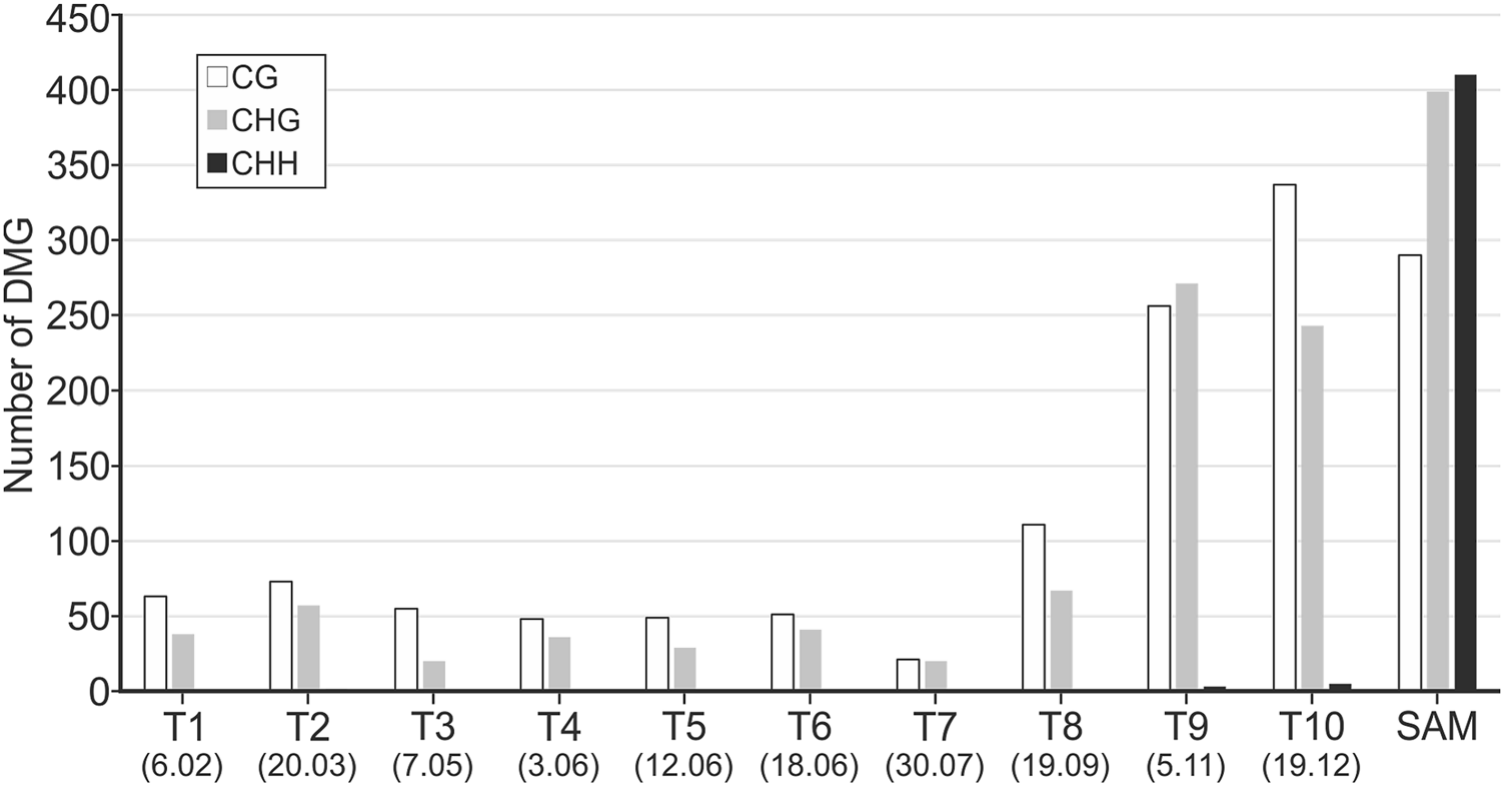
Number of differentially methylated genes among 10 time points (T1–T10, dates in parentheses) throughout the year in buds/shoot tips of clonal Norway spruce WE and CE (clone B10V) epitype trees and shoot apical meristems (SAM) of corresponding clonal mature somatic CE and WE embryos. For each epitype, three individual trees were analyzed per time point, and three embryo samples, each consisting of 5 embryos, were analyzed

The embryos shared a substantial number (around 45) of DMGs with buds of epitype trees mostly during the early-winter period at T9–T10 (November–December) (Suppl. Fig. S3). Similarly, the number of DMGs for the CG and CHG contexts was comparable between the epitype apical parts of embryos and epitype trees at T9–T10 (Fig. 10). In the CHH context, there were close to no-stable DMGs throughout the annual cycle, while in the embryos, more than 400 were reported (Fig. 10, Viejo et al. 2023). For all other time points, there were considerably less DMGs (around 6–14) shared between SAM of somatic embryos and trees.

Among the 30 most stable DMGs through the annual cycle, 22 had similar trends of DNA methylation as previously reported for the embryos, whereas 8 DMGs showed opposite trends. The highest number of retained DMGs was among the epigenetic machinery-related genes (Suppl. Table S1.1).

Analyses of the distribution of specific DMRs across the studied genomic regions of the six stable DMGs through the annual cycle in the epitype trees revealed similar DMRs in the CG context as in embryos (Table S2). Three of these genes were hyper-methylated in the CE epitype relative to the WE both in trees and SAMs of embryos: MA_159389g0010 (*PaAGO2*) in the 1022–1129 and 1138–1324 bp regions of the analyzed promoter sequence, MA_27101g0010 (*PaCRK42*) in the 882–998 bp region and MA_117712g0010 (*PaAP1*) in the 1353–1479 bp region (Suppl. Table S2). For the *PaAGO7* gene (MA_10425786g0020) that was hyper-methylated in the WE epitype trees relative to CE, similar DMRs were found in RAM but not SAM part of WE epitype embryos.

## Discussion

The temperature experienced during post-meiotic megagametogenesis (zygotic embryogenesis) and somatic embryogenesis can induce epigenetic memory resulting in long-lasting bud phenology changes in the progeny (Johnsen et al. 2005a, b; Kvaalen and Johnsen 2008; Carneros et al. 2017). In the gymnosperm Norway spruce, contrasting temperature sums during somatic embryogenesis were shown to create clonal epitypes differing in their bud phenology in a predictable and reproducible manner (Kvaalen and Johnsen 2008; Carneros et al. 2017; Fossdal et al. 2024). Here we report for the first time stable and shifted DNA methylation differences between clonal Norway spruce CE and WE epitype trees that have retained different bud phenology traits after more than a decade in common garden conditions. This suggests that both stable and temporary shifted epigenetic markers are associated with climatic memory. Moreover, comparisons between the clonal epitype trees and the corresponding clonal epitype embryos revealed similar DMRs in specific DMGs, indicating DNA methylation is involved in establishing and maintaining this epigenetic memory.

The detailed common garden observations conducted in the spring and early summer showed a distinct epigenetic difference in the bud burst-timing of about 8 days between the clonal CE and WE epitype trees (Fig. 2). This clearly demonstrates that the long-term stable epigenetic memory is retained after many years under field conditions. These findings extend and support previous, less detailed, phenological observations on epitypes of the same clone at the seedling and young sapling stage (6 years old) (Kvaalen and Johnsen 2008; Carneros et al. 2017). The detailed recordings in the current study revealed that the period of significantly different phenological development between the epitypes during the spring and early summer lasted for about one month, covering most of the period from the bud swelling, through the bud burst and shoot elongation to the new bud set (Fig. 2). Although the WE lagged behind in bud burst, regrowth, and new bud set, both epitypes had new buds formed already on June 16, consistent with bud set occurring under long days in mature trees in contrast to seedlings where bud set is induced by short days (Olsen 2010).

Conceptually, epigenetic memory in plants is a complex interplay of DNA methylation, histone modifications, sRNAs, RNA-mediated regulation, and other epigenetic mechanisms (Yakovlev et al. 2012). DNA methylation is one of the most studied epigenetic marks, which can regulate genome functioning and induce plant resistance and adaptation to abiotic stresses (Ito et al. 2019; Sun et al. 2022; Peck and Sork 2024). In the gymnosperm maritime pine, DNA methylation marks induced during embryogenesis are transmitted to the postembryonic plant development phase (Trontin et al. 2025). In that study, they found genes involved in defense, adaptation, embryo development, and chromatin regulation as candidates for the establishment of a persistent epigenetic memory in 2-year-old trees. Woefully, their maritime pine trial suffered from large damages from wild animals, so the actual epigenetic impact on phenotype beyond the first 36 months was undetermined (Trontin et al. 2025). It has also been reported that the natural variation of DNA methylation may reflect local adaptations of Scots pine populations (Alakärppä et al. 2018). In Norway spruce, epitype-inducing temperature conditions during embryogenesis inducing epigenetic memory involve specific DNA methylation changes (Viejo et al. 2023). In the resulting WE and CE epitype embryos, genes related to epigenetic machinery, such as DNA methyltransferases and *ARGONAUTEs*, were differentially methylated. In this study, we examined whether the observed epigenetic memory is associated with stable or temporary shifted (in a clock-like fashion) DNA methylation mark differences in shoot apices between the CE and WE epitype trees as the seasons advance, using targeted bisulfite sequencing at a single-base resolution for 2744 selected target genes involved in the epigenetic machinery, timing of phenology, development, and the circadian clock. The same setup was recently used to identify differences in DNA methylation between CE and WE epitype embryos (Viejo et al. 2023).

Cluster analysis, comparing the global DNA methylation levels in the different methylation contexts in all genic regions for all time points through the year, confirmed clear differentiation between the epitype trees for the CG context (Figs. 4, S2). In the CHG context, the differentiation between the epitypes was not fully consistent, while CHH methylation differences showed a random pattern between epitypes. In the CG context, cluster analysis not only separated the epitype trees but also most samples from the same individual tree within each epitype. Despite the high variability of methylation marks during annual development, there is canalization of placement of methylation marks between the epitypes, represented in the CG context and less robust for CHG context and very unstable for CHH contexts. This might reflect that the induced DNA methylation differences tend to be inherited in the CG context over many mitotic generations but less so for CHG and not at all for the CHH, possibly reflecting that the methylation maintenance mechanism for CG (concomitant with replication) is very effective, while less so for the CHG (dependent on H3K9 di-methylation), while CHH marks (dependent on RdDM pathway) show no signs of maintenance at all in these 2744 genes after more than a decade in common garden conditions.

Distribution of methylated cytosines, along the studied promoter and gene body (first exon and part of first intron) regions, was similar to that reported in other plant species, including trees (Ausin et al. 2016; Zhang et al. 2020; Li et al. 2023). Specifically, the promoter regions of the 2744 selected genes tended to have higher DNA methylation levels than the coding part in the gene bodies with a progressive reduction in DNA methylation along the 2 kb promoter sequence toward the TSS, and stably low DNA methylation level along the first 1000 bp of the coding region examined (Fig. S2). The highest number of differentially methylated cytosines between epitypes was found in the promoter region, the lowest in the area preceding the TSS, and a local maximum in the middle of the coding region included (Fig. 8). Thus, the majority of epitype-specific DNA methylation/demethylation differences occur between 1500 and 750 bp of the promoter and 400 and 800 bp after the TSS. Considering that the methylation status of gene promoters is proposed to impact gene expression variation during plant development and growth (Brenet et al. 2011; Lucibelli et al. 2022), the differential methylation of promoters in the targeted genes between the CE and WE trees may be a feature of epigenetic memory. DNA methylation of the first exon has been linked to reduce transcript levels and transcriptional silencing (Muyle et al. 2022). Epigenetic marks in the first intron may also have post-transcriptional effects on gene expression (Zalabák and Ikeda 2020).

Multiple studies have shown that DNA methylation levels are actively regulated by plant during growth, development, and throughout a plant’s life cycle, including response to stress, reflecting an important role for DNA methylation (Bartels et al. 2018; Zhang et al. 2018; Ito et al. 2019; Bennett et al. 2021; Kumar and Mohapatra 2021; Shaikh et al. 2022). Consistent with this, the overall DNA methylation levels in the set of 2744 genes in the epitype trees through the annual cycle revealed higher methylation levels in the CG and CHG contexts during growth and lower methylation levels during dormancy (autumn, winter, early spring) (Fig. 4). Seasonal patterns of DNA methylation were reported in natural populations of *Arabidopsis halleri*, showing different patterns among all contexts. In the CG context, the highest methylation levels were then observed during summer, for CHG in March and late autumn, and for CHH during autumn (Ito et al. 2019). Notwithstanding, the largest differences in DNA methylation between the CE and WE epitypes were found in November–December and June (Fig. 4).

Differences in the dynamics of DNA methylation patterns between epitypes were visualized using polar area diagrams. For both the CG and CHG contexts, most methylation differences occurred during the dormant period in November–December but multiple hyper- and hypo-methylated DMRs were also detected during June (Fig. 5). Taken together, there are apparently two pivotal dates during the yearly cycle, with the highest levels of DNA methylation differences between epitypes; June and November–December, which correspond to the summer and winter solstices. It is well known that the circadian clock coordinates developmental, physiological, and metabolic processes in plants with changes in day length, light, and temperature throughout the year and warming (Muranaka et al. 2022; Oravec and Greenham 2022). Also, a relationship between leaf senescence and warming before and after solstice has been shown for a wide range of forest tree species (Zohner et al. 2023). Therefore, DNA methylation dynamics seems to be involved in controlling crucial physiological responses in plants to environmental signals.

Temporal distribution of stable DMRs in genes indicates that most of the stable differential methylation occurred during the dormant period during September–March. Only 2 genes, MA_10433153g0010 and MA_94930g0010, possessed stable methylation marks also in May–July, just before and after bud burst. Both genes contain penta- or tetra-tricopeptide repeat domains and are involved in the regulation of gene expression on the RNA and protein levels (Barkan and Small 2014; Manna 2015). Interestingly, two *ARGONAUT*-like genes, *PaAGO2* (MA_159389g0010) and *PaAGO7* (MA_10425786g0020), were stably differentially methylated throughout the whole year. *PaAGO2* was stably hyper-methylated in CE and *PaAGO7* in WE. These give rise to transcripts with CDS length of 690 and 714 bp, respectively. *ARGONAUT*-like genes play key roles in the RNA interference pathway that regulates gene expression at the post-transcriptional level by using small RNA molecules to target and silence complementary mRNA sequences (El-Sappah et al. 2021). These observations are consistent with previous results by Yakovlev and Fossdal (2017), who showed highly dynamic miRNA reprogramming in Norway spruce during somatic embryogenesis at different epitype-inducing temperatures.

Our previous study of Norway spruce CE and WE somatic embryos of the same clone revealed 1850 differential DNA methylation patterns between the epitypes in at least one methylation context (CG, CHG, CHH) (Viejo et al. 2023). Here, epitype trees revealed 1548 differentially methylated sites in 479 DMGs. From this number of DMGs, 30 genes were hyper-/hypo- differentially methylated at least seven consecutive times during the year.

Based on our earlier study (Viejo et al. 2023), 68% of the analyzed genes displayed differential DNA methylation patterns between contrasting epitype embryos of the same clone in at least one methylation context (CG, CHG, CHH). This indicates that the epitype-inducing temperature conditions establish an epigenetic memory with epitype-specific DNA methylation changes in Norway spruce (Viejo et al. 2023). A similar observation was recently reported in maritime pine (Trontin et al. 2025). As mentioned above, our epitype trees were produced from the somatic embryos of the same B10V genotype, and were raised in common garden conditions, where they show clear phenotypic differences in terms of phenological characters (Fig. 2; Kvaalen and Johnsen 2008; Carneros et al. 2017). As we used the same set of genes and targeted BS approach, we compared the current data on methylation patterns in adult trees through the annual cycle with the methylation patterns in corresponding somatic embryos. Specifically, we compared the SAM part of the embryos with the SAM-containing shoot tips/buds. We refer to all our comparisons here just for the SAM part of embryos. The overall DNA methylation levels for the CHG and CG contexts were relatively similar between SAMs of embryos and adult trees of the epitypes at least during late autumn/early winter. However, there was a drastic decrease in CHH methylation levels in the adult trees compared to the somatic embryos. CHH is a more "labile" mark and more difficult to maintain after cell division due to its non-symmetrical nature. This appears similar to *Arabidopsis*, where the relatively stable CG and CHG methylation levels were observed after germination compared to dry seeds, as well as extensive gain of CHH methylation during seed development and drastic loss of CHH methylation during germination (Kawakatsu et al. 2017). In general, the embryo methylomes were characterized by a higher number of DMGs between epitypes than in shoot tips/buds of adult epitype trees. The highest number of DMGs in buds that were comparable with embryonic SAMs was detected only in late autumn–winter (November–December), when most methylation/demethylation events occurred. Thus, despite the lack of visible growth, the plants are apparently and actively sensing the environment and preparing for the next growing season. The presence of epigenetic modifications related to the environmental conditions affecting dormancy has previously been reported in many fruits and forest trees (Rios et al. 2014; Lloret et al. 2018; Prudencio et al. 2018; Rothkegel et al. 2020). Also, the predetermined growth observed in mature trees, including Norway spruce, may potentially at least partly be associated with epigenetic modulations of gene expression (Logan and Polard 1975). We established partial memory transition effects by analyzing the relations between DMRs in embryo SAMs and shoot tip/buds. Out of the six candidate genes with stable DNA methylation pattern throughout the year, four genes had the same DMRs in the CG context in SE. Three genes (*PaAGO2*, *PaCRK42*, and *PaAP1*) were hyper-methylated in CE and *PaAGO7* was hyper-methylated in WE, in SAM of embryos as well as buds. These gene models should be further studied as putative epitype markers. Two genes that were differentially methylated in the CHG contexts had DMRs in different areas in different points that did not always correspond to DMRs in the SAMs of embryos. Similar findings have been shown for somatic embryos and shoot tips of 2-year-old seedlings of maritime pine (Trontin et al. 2025). In that study, it was demonstrated not only that DNA methylation patterns are established in response to temperature during embryogenesis, but also that they are partially retained in plant SAM (Trontin et al. 2025).

In conclusion, DNA methylation analysis of Norway spruce epitypes throughout the annual cycle showed that the differences in methylation of cytosines in different contexts were very dynamic and greatly varied in different annual developmental stages. Despite this, we found clear differences in methylation patterns between the epitype and the existence of specific circannual rhythms affecting methylation levels for the studied genomic regions. We defined two “hot spot” time points of DNA methylation rearrangements related to winter and summer solstices: one common for both epitypes in December and one just for CE in June. These results indicate that the most active DNA methylation changes occur during dormancy period (methylation/active demethylation). It appears that December (and maybe January, where sampling was not performed) is very important for establishing DNA methylation patterns for the next growing season. We identified the stable methylation marks in the promoter regions of specific genes, related to regulation of gene expression, differentially methylated between epitypes during specific time periods and putative embryo–adult transmission for some genes, indicating that the DNA methylation marks in genomic regions throughout the annual cycle may be part of an induced epigenetic memory in embryos that is later manifested as phenologically different epitype trees. Most stable methylation marks were identified among CG contexts, less in CHG, and nothing in CHH contexts, which could reflect the inheritance mode of different methylation contexts.

## Supplementary Information

Below is the link to the electronic supplementary material.

Supplementary file1 (DOCX 2943 KB)

Supplementary file2 (PDF 511 KB)

Supplementary file3 (XLSX 159 KB)

Supplementary file4 (XLSX 25 KB)

Supplementary file5 (XLSX 1341 KB)

## Data Availability

The data discussed in this publication have been deposited in NCBI’s Gene Expression Omnibus (Edgar et al. 2002) and are accessible through GEO Series accession number GSE222491 (https://www.ncbi.nlm.nih.gov/geo/query/acc.cgi?acc = GSE222491).

## Abbreviations

CE: Cool epitype
DMG: Differentially methylated gene
DMR: Differentially methylated region
DMS: Differentially methylated site
WE: Warm epitype
SAM: Shoot apical meristem
TSS: Transcription start site
