# Supplementary material for: Stable and temporally shifted DNA methylation differences between 14-year-old Norway spruce epitypes under common garden conditions
Source: Planta. 2026 Aug 7;264(3):88. doi: 10.1007/s00425-026-05116-6 (PMC13451259; doi:10.1007/s00425-026-05116-6)
Supplement: Supplementary file 1 [file 425_2026_5116_MOESM2_ESM.pdf]

Suppl. Fig. S4 Specific locations (positions) of the differentially methylated sites (DMS) where the addition (**A**) or removal (**B**) of a methyl group in CE significantly varies between epitypes for the selected genes. List and description of selected genes is presented in the table after the graphs

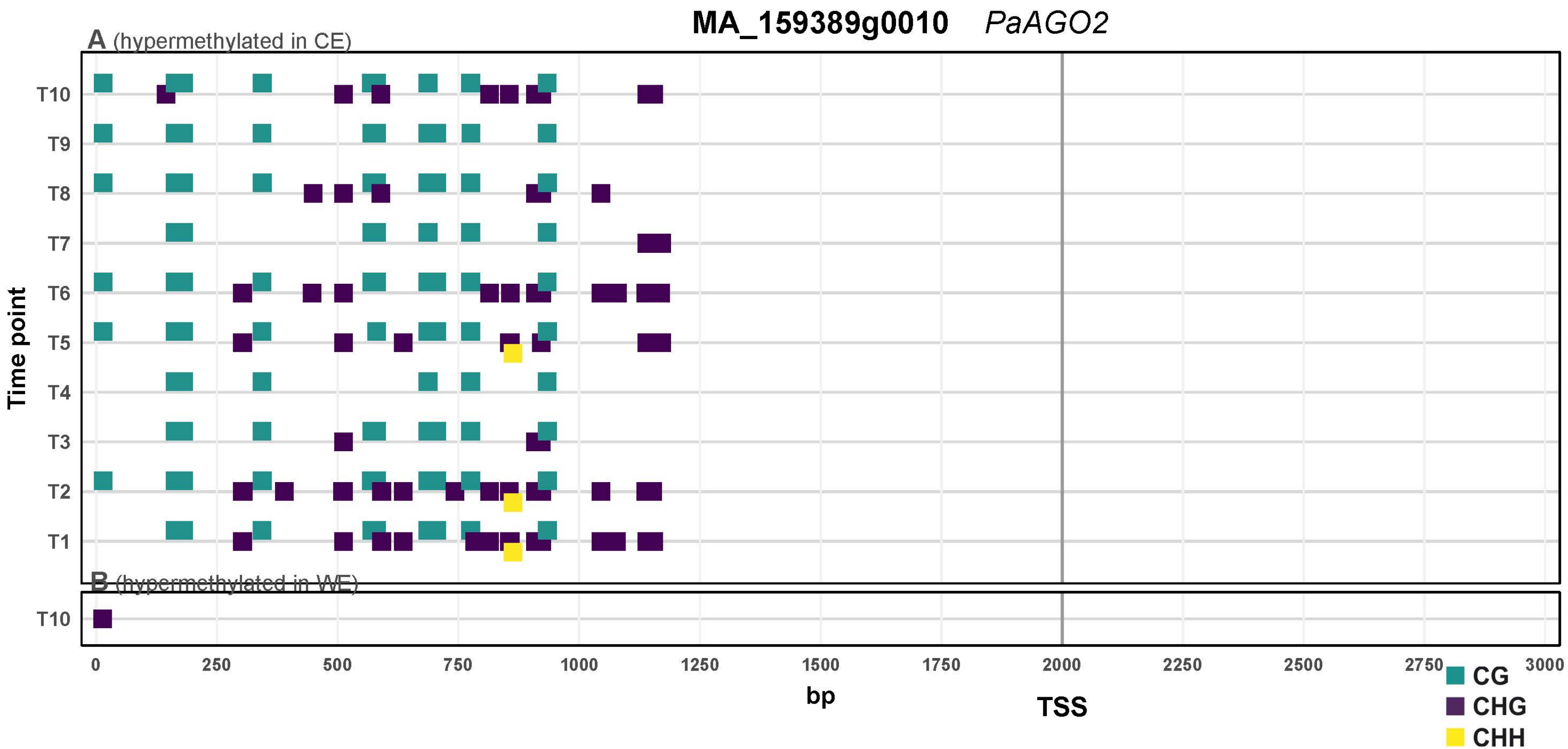

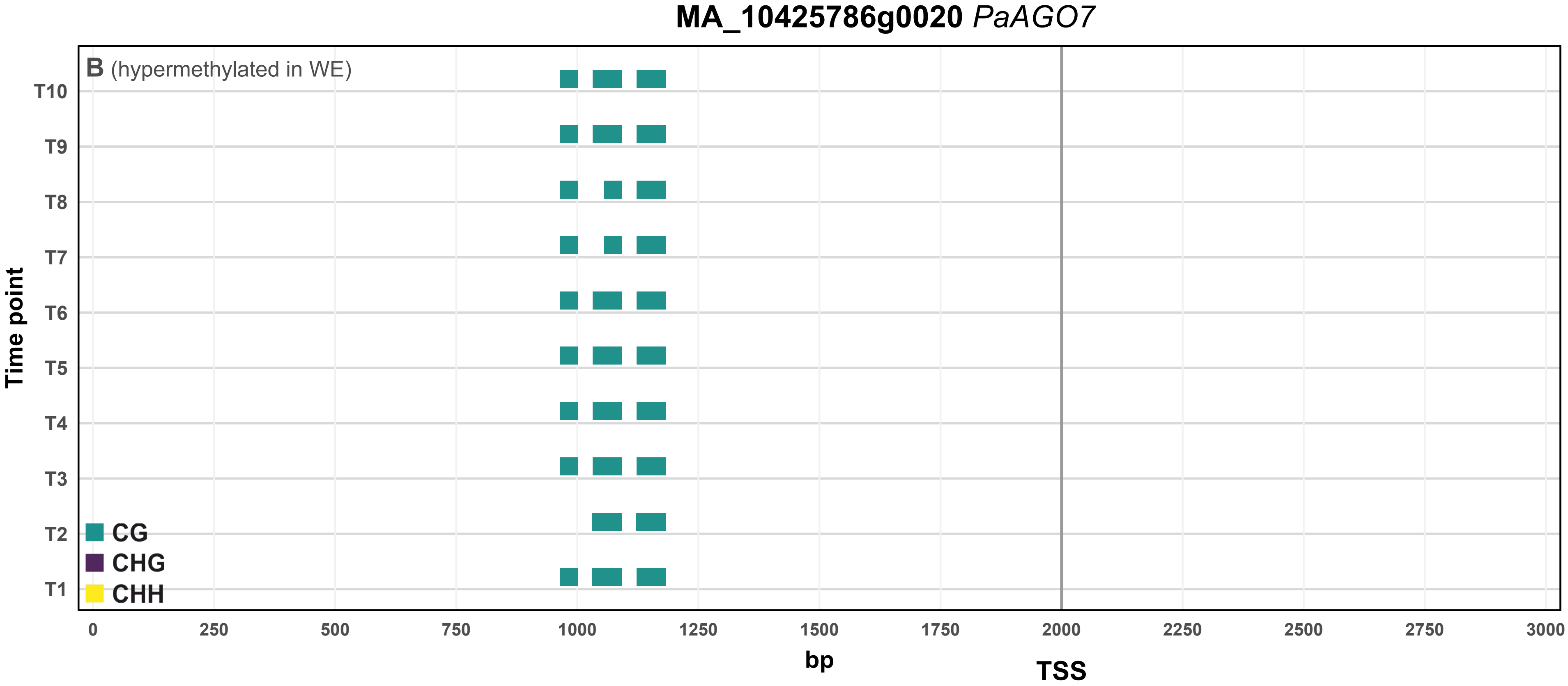

MA_10425786g0020 *PaAGO7*
B (hypermethylated in WE)
Time point
T10
T9
T8
T7
T6
T5
T4
T3
T2
T1
CG
CHG
CHH
0
250
500
750
1000
1250
1500
1750
2000
2250
2500
2750
3000
bp
TSS

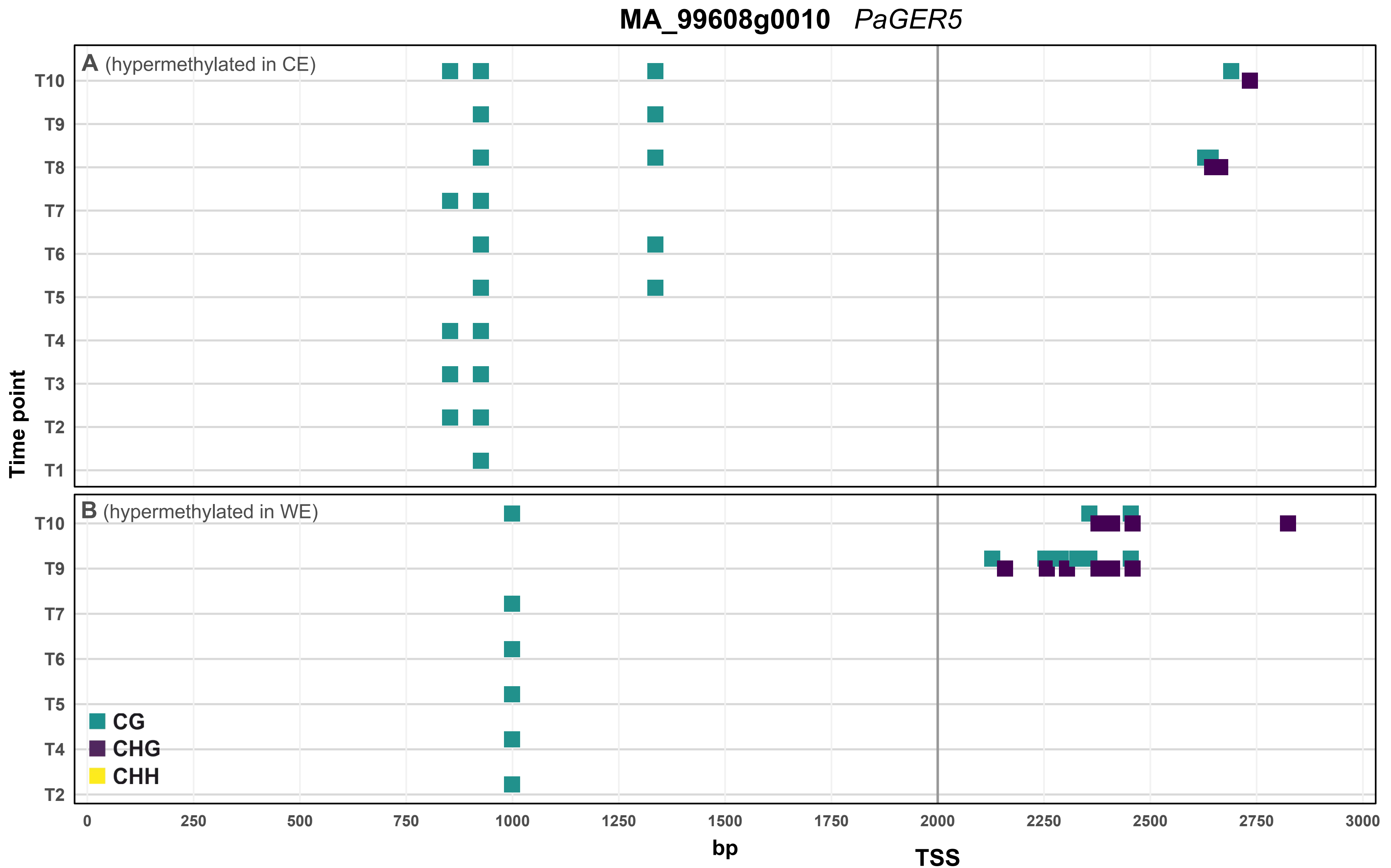

MA_99608g0010 PaGER5
A (hypermethylated in CE)
B (hypermethylated in WE)
T10
T9
T8
T7
T6
T5
T4
T3
T2
T1
Time point
CG
CHG
CHH
0
250
500
750
1000
1250
1500
1750
2000
2250
2500
2750
3000
bp
TSS

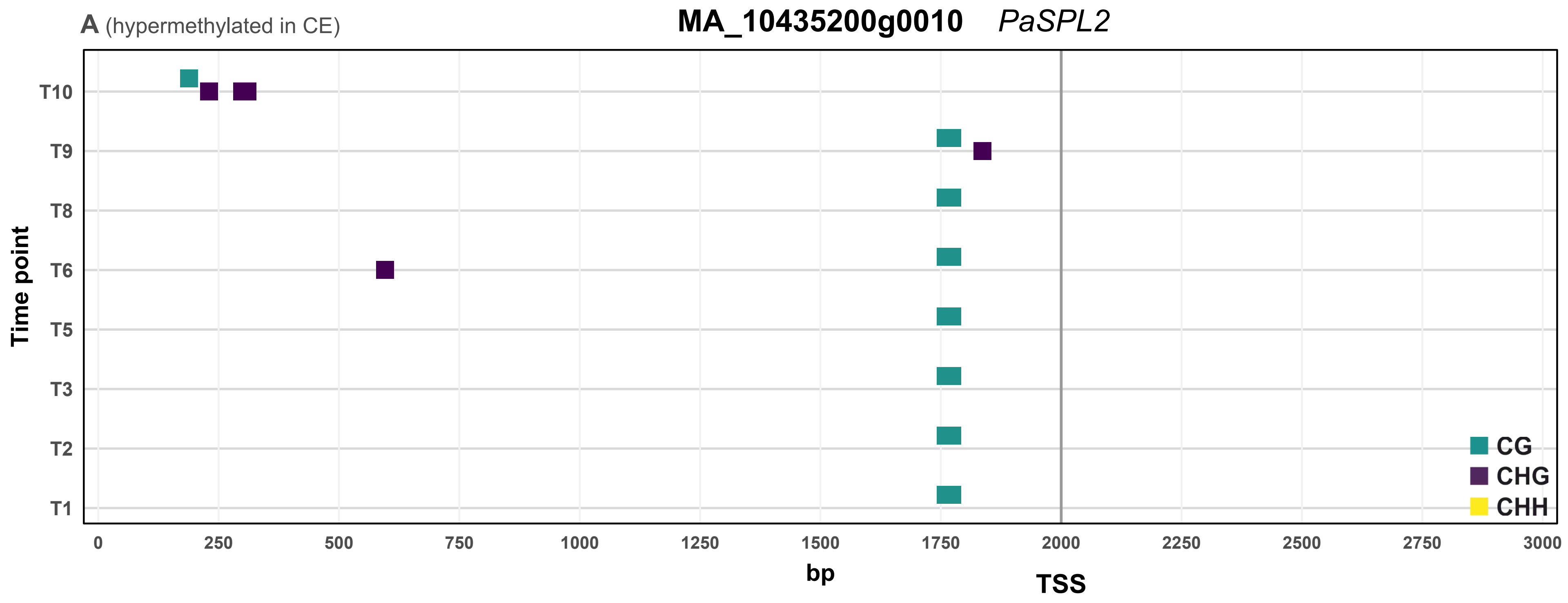
A (hypermethylated in CE)
MA_10435200g0010 PaSPL2
Time point
T10
T9
T8
T6
T5
T3
T2
T1
0
250
500
750
1000
1250
1500
1750
2000
2250
2500
2750
3000
bp
TSS
CG
CHG
CHH

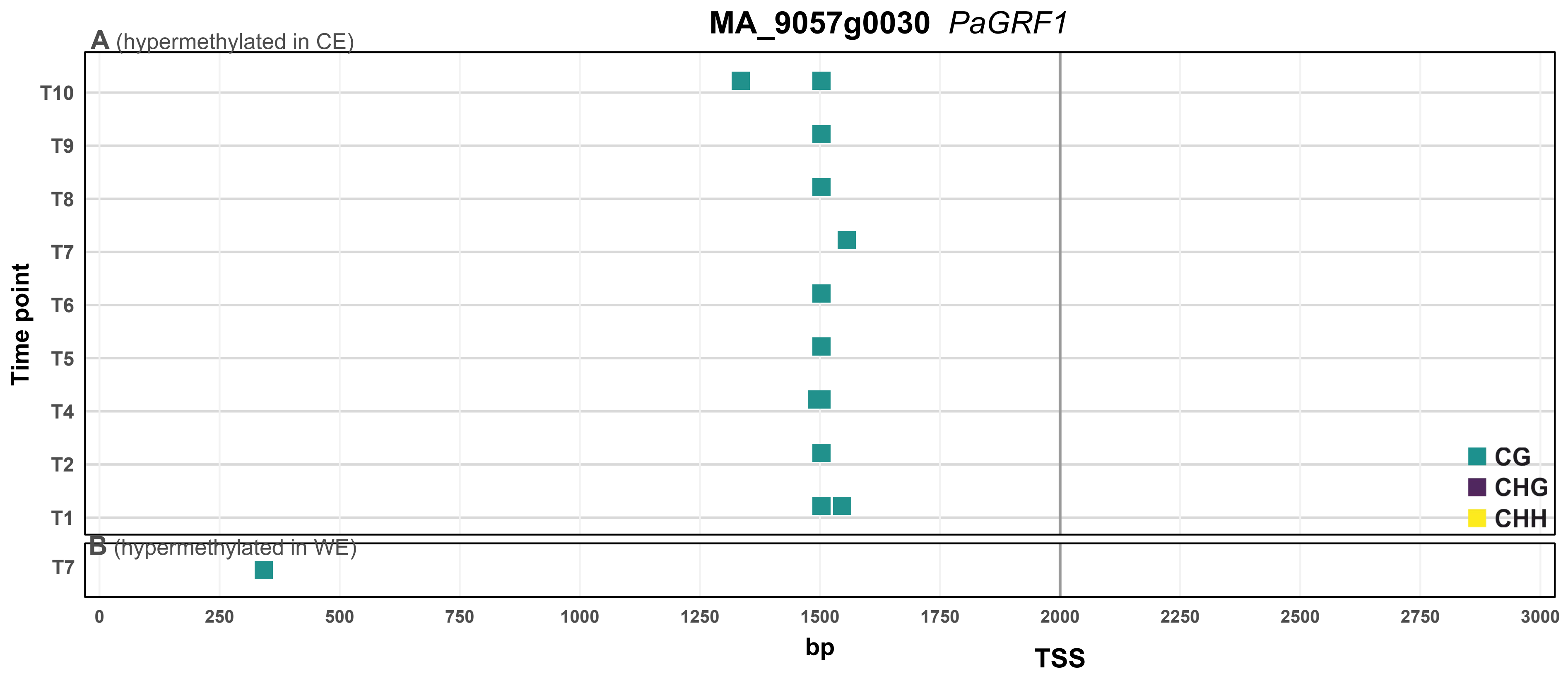

MA_9057g0030 PaGRF1
A (hypermethylated in CE)
B (hypermethylated in WE)
Time point
T10
T9
T8
T7
T6
T5
T4
T2
T1
T7
CG
CHG
CHH
0
250
500
750
1000
1250
1500
1750
2000
2250
2500
2750
3000
bp
TSS

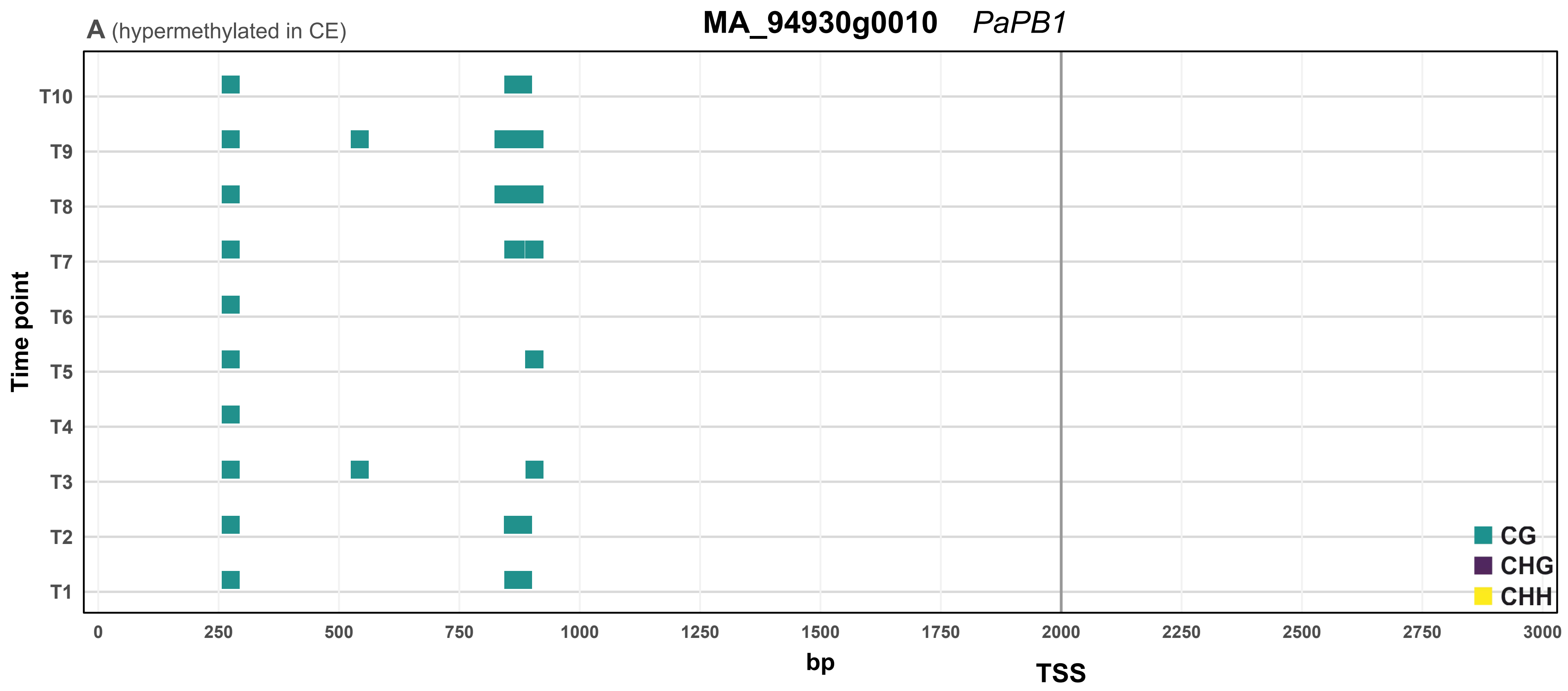
A (hypermethylated in CE)
MA_94930g0010 PaPB1
Time point
T10
T9
T8
T7
T6
T5
T4
T3
T2
T1
0
250
500
750
1000
1250
1500
1750
2000
2250
2500
2750
3000
bp
TSS
CG
CHG
CHH

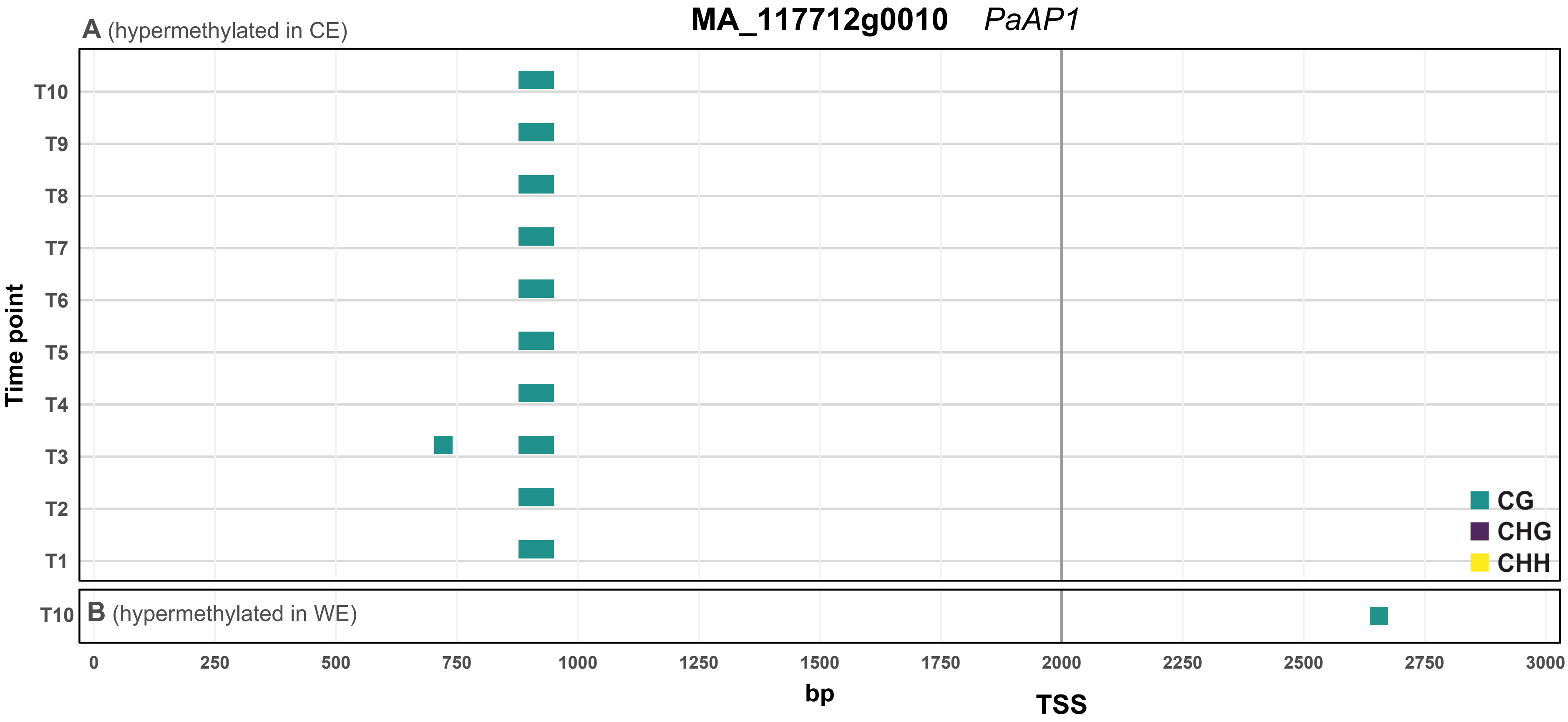
MA_117712g0010 PaAP1
A (hypermethylated in CE)
B (hypermethylated in WE)
Time point
T10
T9
T8
T7
T6
T5
T4
T3
T2
T1
CG
CHG
CHH
0
250
500
750
1000
1250
1500
1750
2000
2250
2500
2750
3000
bp
TSS

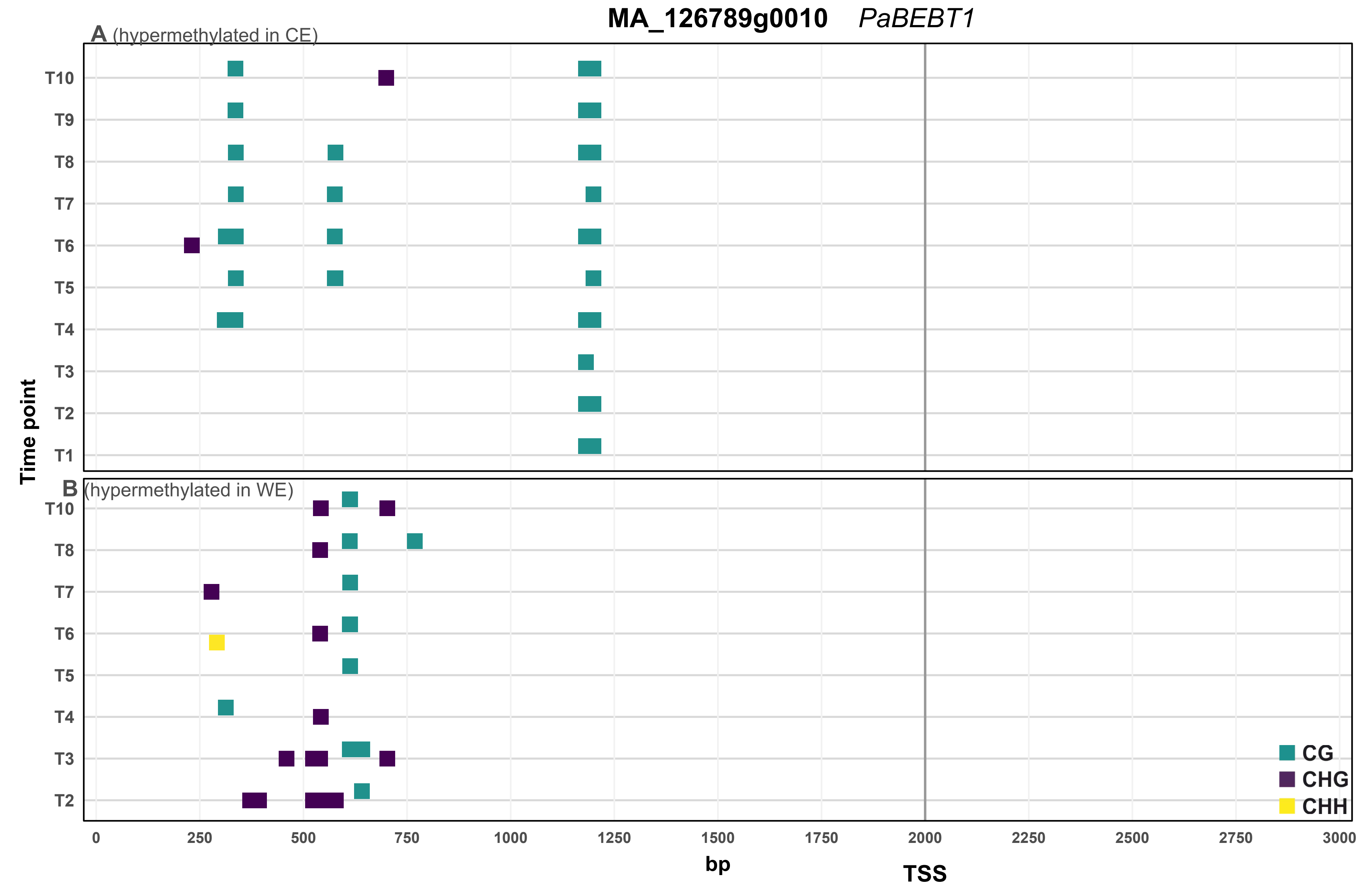
MA_126789g0010 PaBEBT1
A (hypermethylated in CE)
B (hypermethylated in WE)
Time point
T10
T9
T8
T7
T6
T5
T4
T3
T2
T1
0
250
500
750
1000
1250
1500
1750
2000
2250
2500
2750
3000
bp
TSS
CG
CHG
CHH

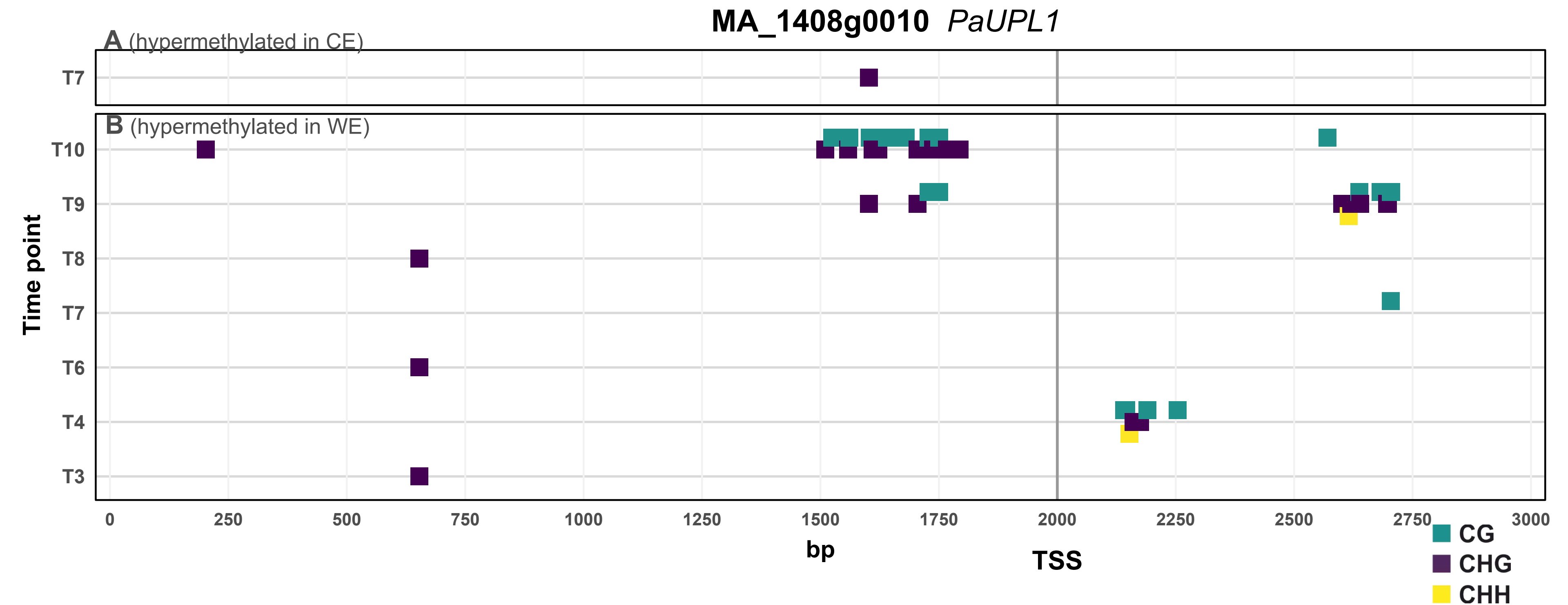
MA_1408g0010 PaUPL1
A (hypermethylated in CE)
B (hypermethylated in WE)
T7
T10
T9
T8
T7
T6
T4
T3
Time point
0
250
500
750
1000
1250
1500
1750
2000
2250
2500
2750
3000
bp
TSS
CG
CHG
CHH

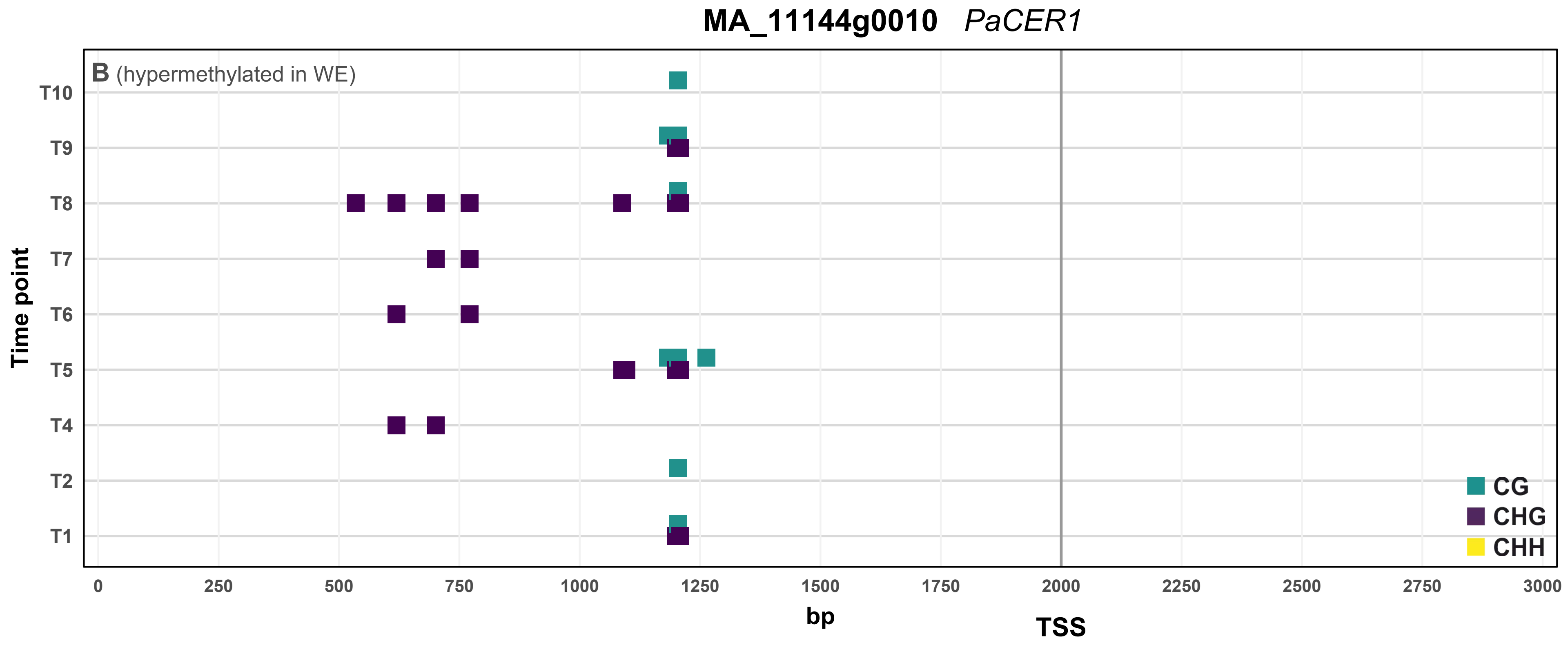

MA_11144g0010 *PaCER1*
B (hypermethylated in WE)
T10
T9
T8
T7
T6
T5
T4
T2
T1
Time point
0
250
500
750
1000
1250
1500
1750
2000
2250
2500
2750
3000
bp
TSS
CG
CHG
CHH

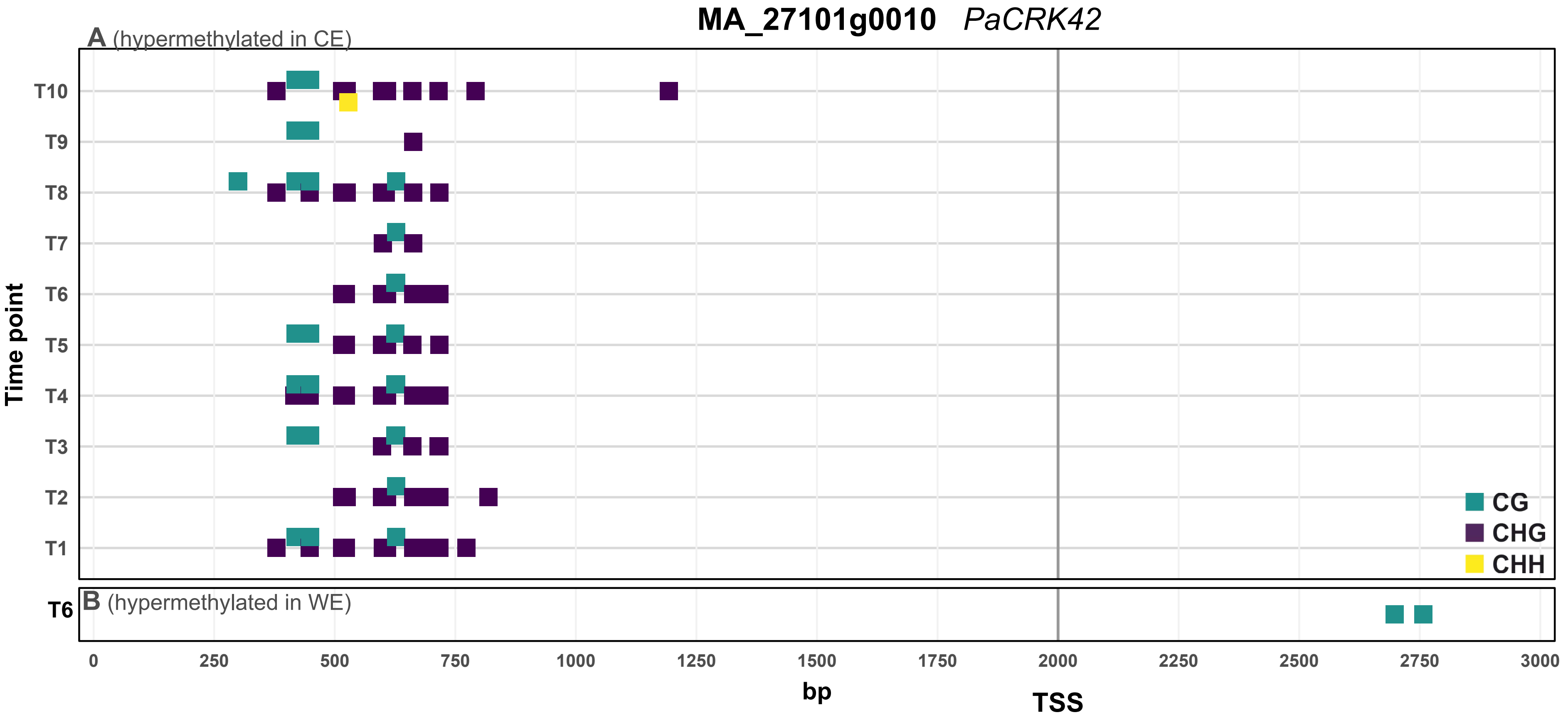

MA_27101g0010 *PaCRK42*
A (hypermethylated in CE)
T10
T9
T8
T7
T6
T5
T4
T3
T2
T1
Time point
CG
CHG
CHH
T6
B (hypermethylated in WE)
0
250
500
750
1000
1250
1500
1750
2000
2250
2500
2750
3000
bp
TSS

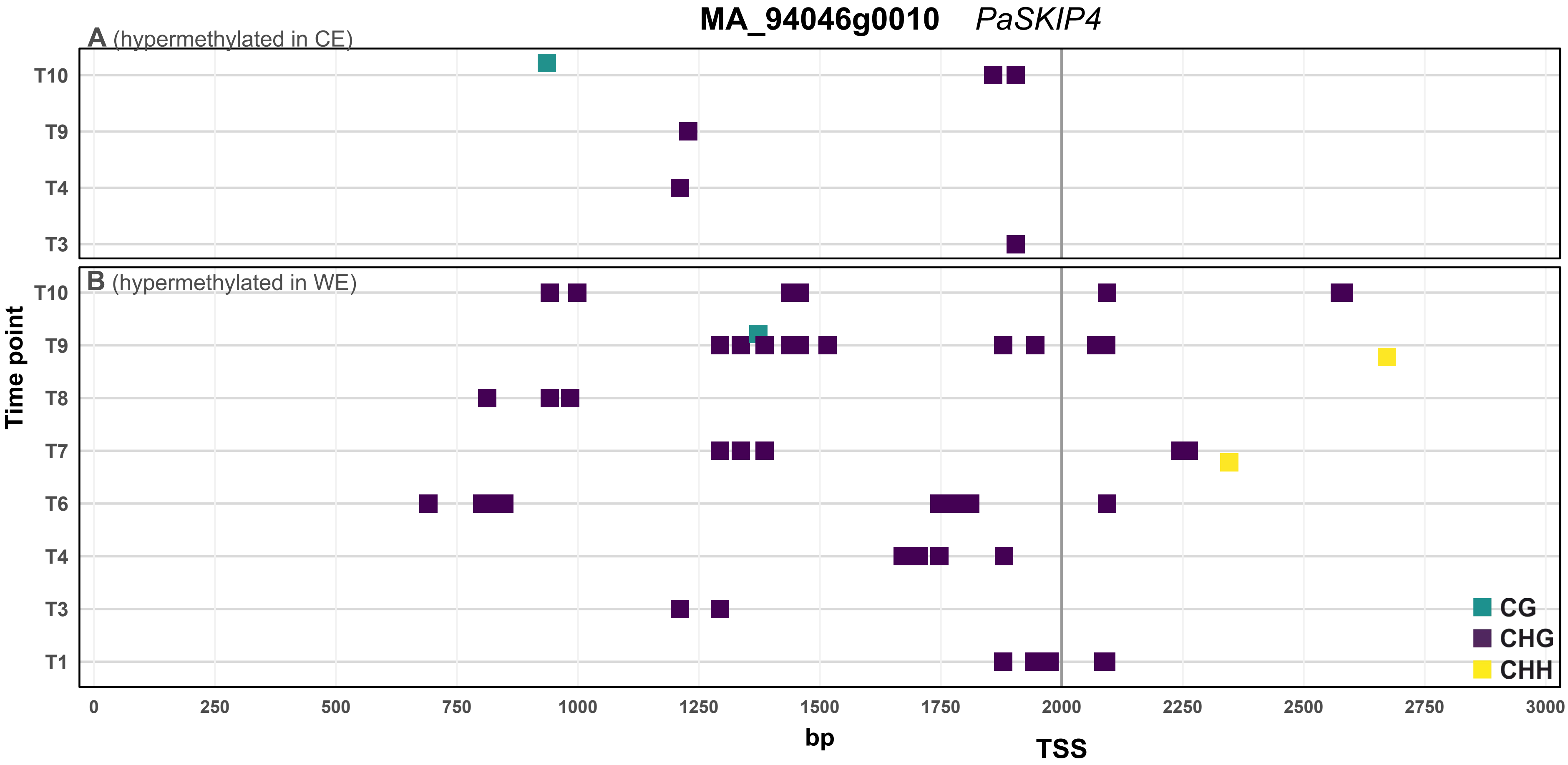
MA_94046g0010 PaSKIP4
A (hypermethylated in CE)
B (hypermethylated in WE)
T10
T9
T4
T3
T10
T9
T8
T7
T6
T4
T3
T1
Time point
0
250
500
750
1000
1250
1500
1750
2000
2250
2500
2750
3000
bp
TSS
CG
CHG
CHH

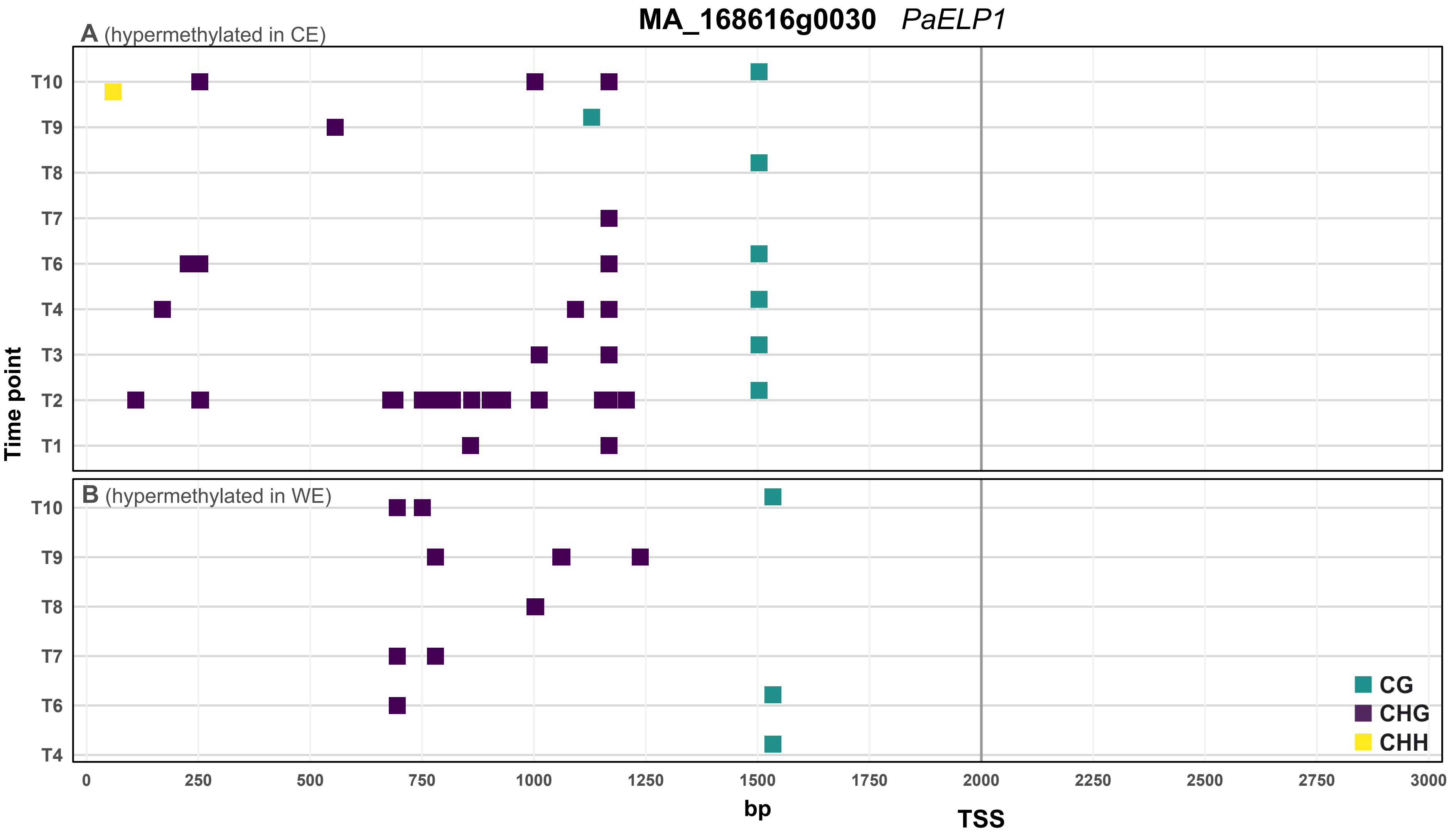

MA_168616g0030 PaELP1
A (hypermethylated in CE)
B (hypermethylated in WE)
Time point
T10
T9
T8
T7
T6
T4
T3
T2
T1
0
250
500
750
1000
1250
1500
1750
2000
2250
2500
2750
3000
bp
TSS
CG
CHG
CHH

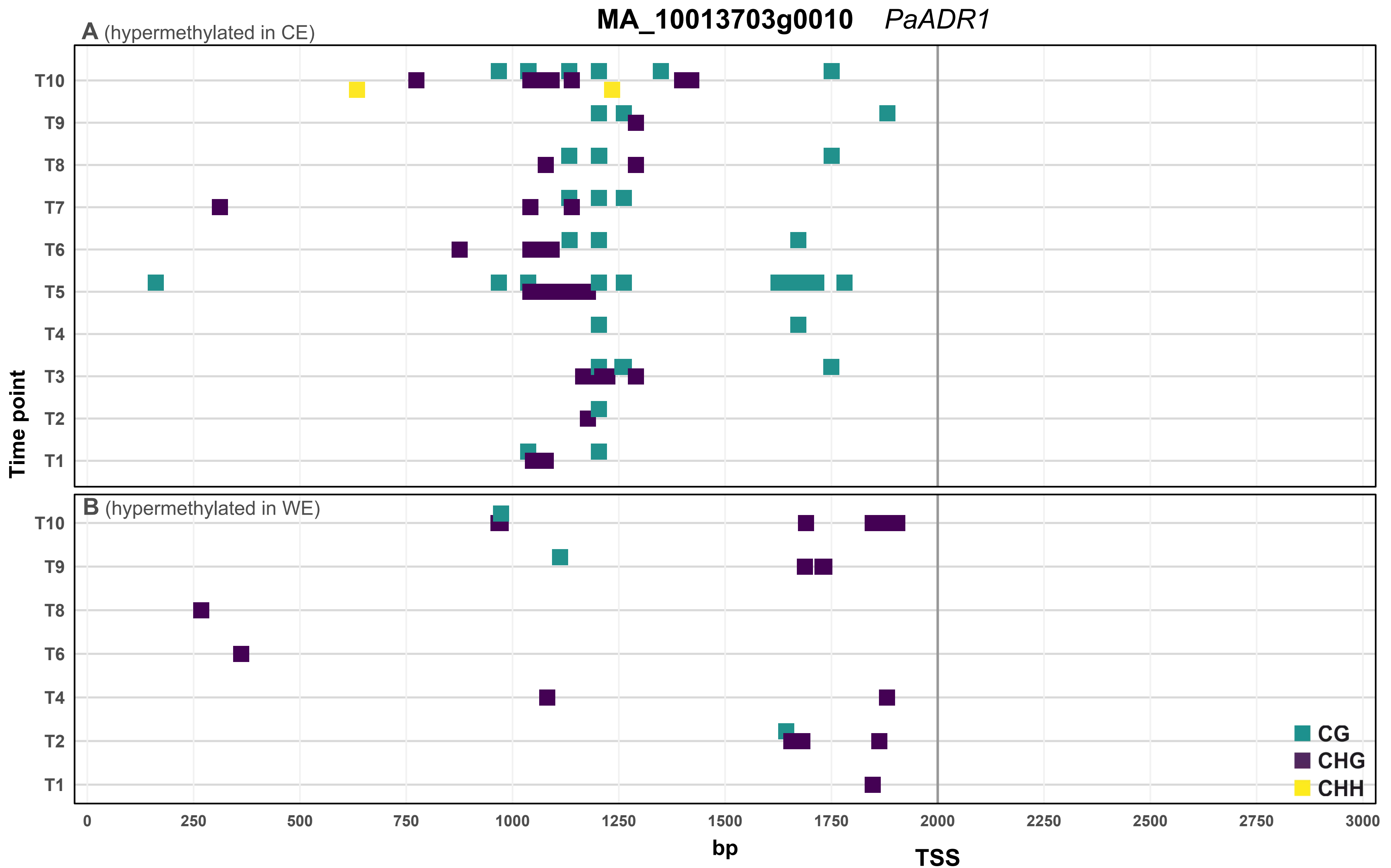
MA_10013703g0010 PaADR1
A (hypermethylated in CE)
B (hypermethylated in WE)
Time point
T10
T9
T8
T7
T6
T5
T4
T3
T2
T1
0
250
500
750
1000
1250
1500
1750
2000
2250
2500
2750
3000
bp
TSS
CG
CHG
CHH

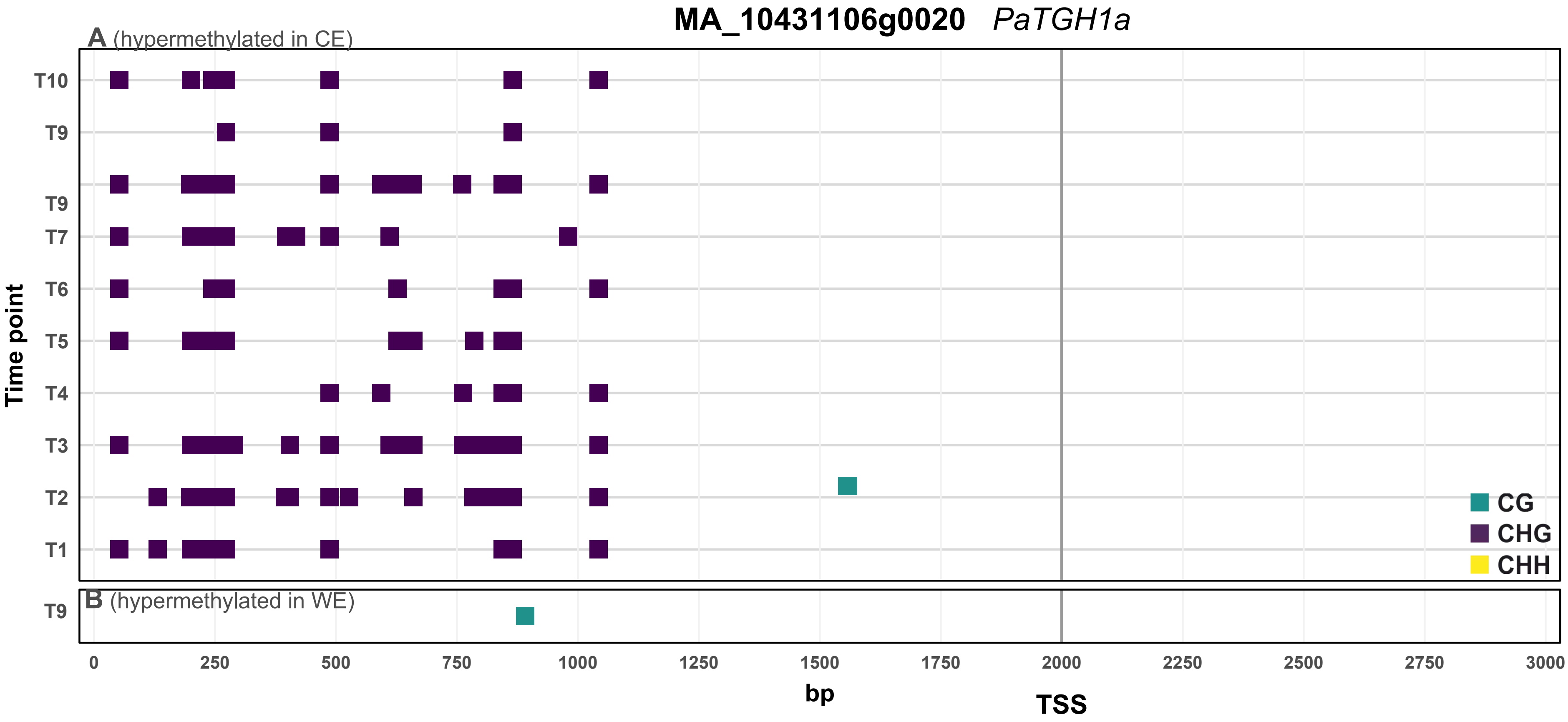

MA_10431106g0020 PaTGH1a
A (hypermethylated in CE)
B (hypermethylated in WE)
Time point
T10
T9
T9
T7
T6
T5
T4
T3
T2
T1
T9
CG
CHG
CHH
0
250
500
750
1000
1250
1500
1750
2000
2250
2500
2750
3000
bp
TSS

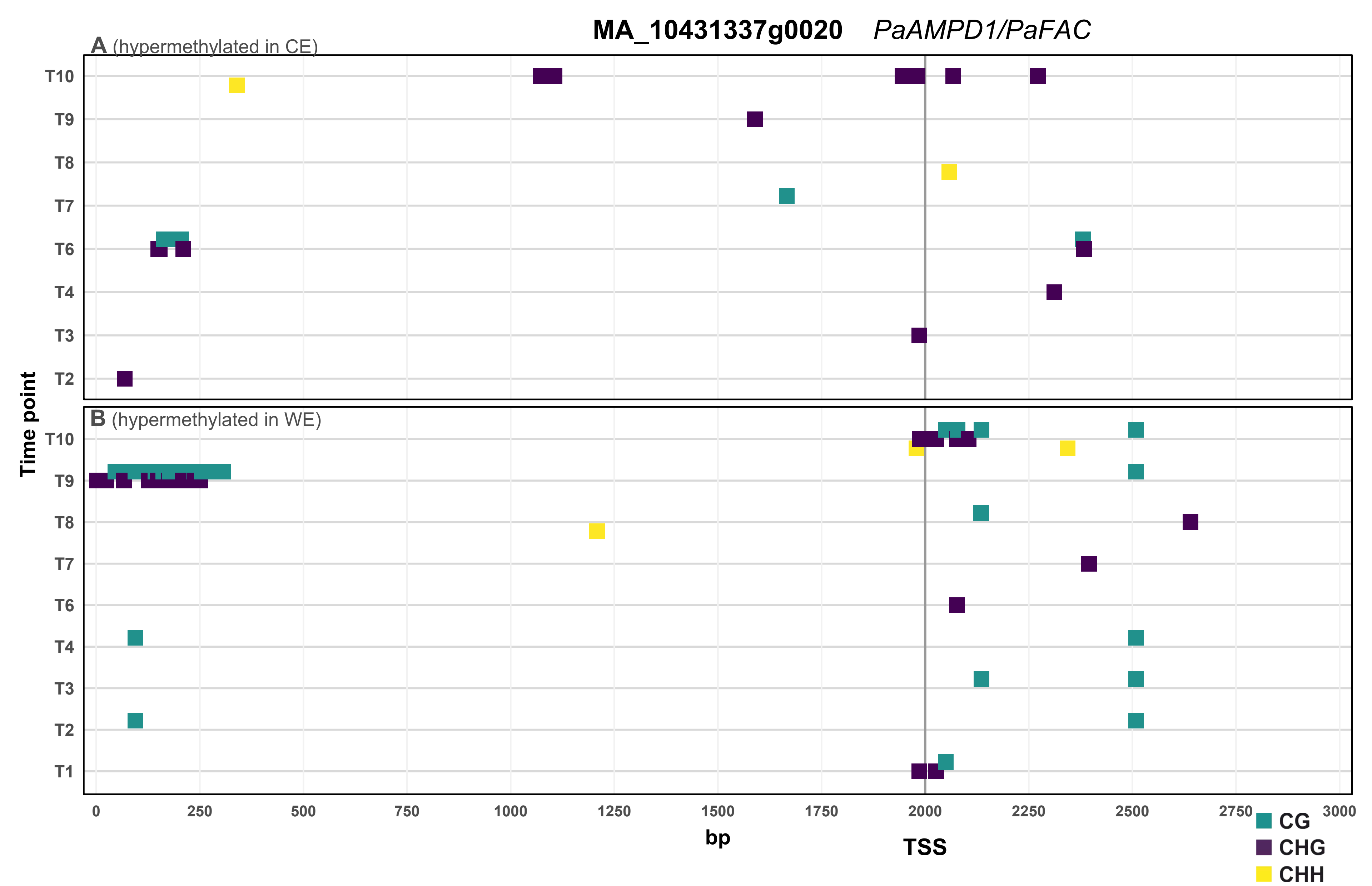
MA_10431337g0020 PaAMPD1/PaFAC
A (hypermethylated in CE)
B (hypermethylated in WE)
Time point
T10
T9
T8
T7
T6
T4
T3
T2
T1
0
250
500
750
1000
1250
1500
1750
2000
2250
2500
2750
3000
bp
TSS
CG
CHG
CHH

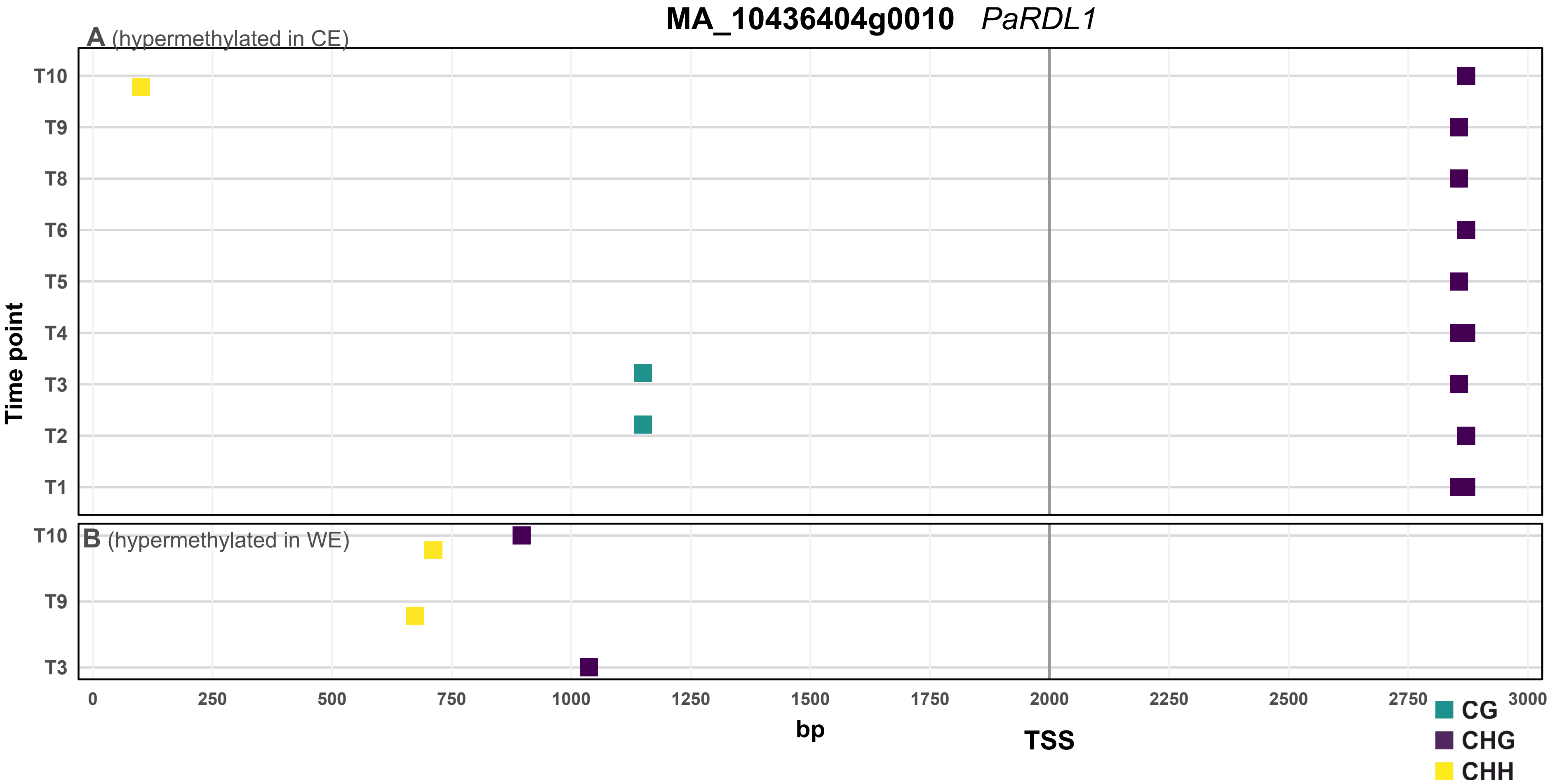

MA_10436404g0010 PaRDL1
A (hypermethylated in CE)
B (hypermethylated in WE)
Time point
T10
T9
T8
T6
T5
T4
T3
T2
T1
0
250
500
750
1000
1250
1500
1750
2000
2250
2500
2750
3000
bp
TSS
CG
CHG
CHH

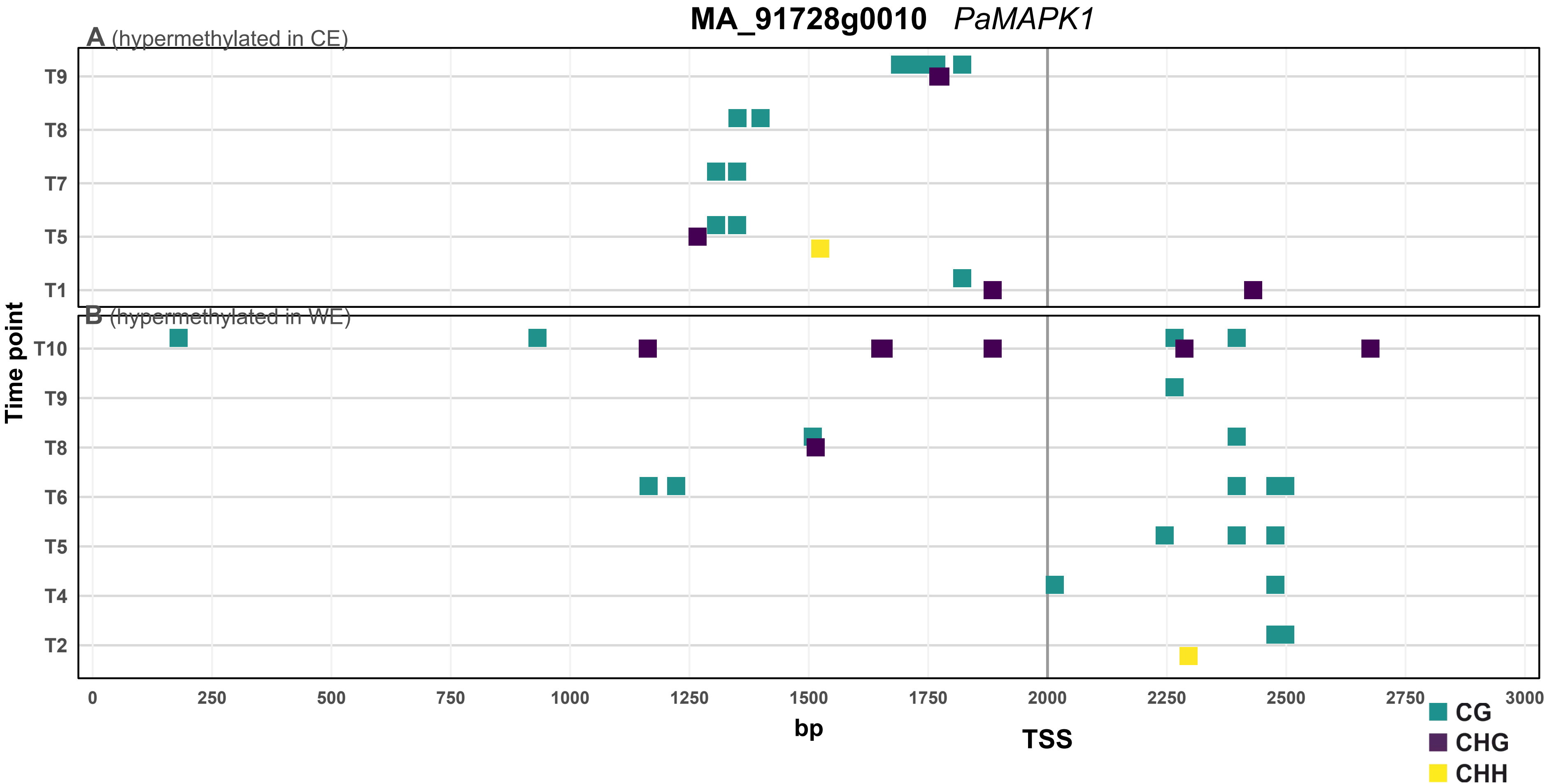
MA_91728g0010 PaMAPK1
A (hypermethylated in CE)
B (hypermethylated in WE)
Time point
T9
T8
T7
T5
T1
T10
T9
T8
T6
T5
T4
T2
0
250
500
750
1000
1250
1500
1750
2000
2250
2500
2750
3000
bp
TSS
CG
CHG
CHH

Gene model ID	Gene name	Pfam annotation	Arabidopsos ortholog	Annotation	Putative functions
MA_10435200g0010	*PaSPL2*	E3 ubiquitin-protein ligase SPL2	AT1G54150.1	RING-type E3 Ubiquitin ligase family protein	SPL2 is a critical regulator of plastid operation and development, controlling how proteins enter the chloroplast
MA_10436404g0010	*PaRDL1*	PF00581-Rhodanese-like domain	AT2G42220.1	Rhodanese/Cell cycle control phosphatase superfamily protein	encode a versatile group of **sulfurtransferase enzymes** essential for sulfur trafficking, cellular redox homeostasis
MA_11144g0010	*PaCER1*	PF04116-Fatty acid hydroxylase superfamily;PF12076-WAX2 C-terminal domain;PF13460-NADH(P)-binding	AT1G02205.3	ECERIFERUM 1-like; CER1, Fatty acid hydroxylase superfamily	The CER1 protein is an integral membrane enzyme located in the endoplasmic reticulum (ER) of epidermal cells. Involved in build the plant's cuticular wax layer
MA_117712g0010	*PaAP1*	PF14541, PF14543-Xylanase inhibitor N-and C-terminals; PF00026-Eukaryotic aspartyl protease	AT1G01300.1	Eukaryotic aspartyl protease family protein	Functions as an anti-cell-death component in reproduction and embryogenesis
MA_126789g0010	*PaBEBT1*	PF02458-Transferase family; aliphatic suberin feruloyl-transferase; ASFT;	AT5G17540.1	Benzyl alcohol O-benzoyltransferase (BEBT); HXXXD-type acyl-transferase family protein	BEBT catalyzes the transfer of a benzoyl group from benzoyl-CoA to an alcohol to form defensive and fragrant volatile esters, such as benzyl benzoate. BEBT uses Coenzyme A (CoA) thioesters as the acyl donor. It facilitates the synthesis of biologically active compounds ranging from floral scents to active metabolites in traditional herbal medicines
MA_27101g0010	*PaCRK42*	PF00069-Protein kinase domain;PF01636-Phosphotransferase enzyme family;PF01657-Salt stress response/antifungal;PF07714-Protein tyrosine kinase	AT5G40380.1	CRK42, CYSTEINE-RICH RLK (RECEPTOR-LIKE PROTEIN KINASE) 42	CRK42 acts as a cellular sensor crucial for plant immunity, reactive oxygen species (ROS) signaling, and abiotic stress responses
MA_9057g0030	*PaGRF1*	PF00098-Zinc knuckle; PF06839-GRF zinc finger	AT5G13920.1	GRF (GROWTH-REGULATING FACTOR) zinc finger / Zinc knuckle protein	The growth-regulating factor (GRF) family of transcriptional factors are involved in the control of leaf size and senescence, inflorescence and root growth, grain size, and plant regeneration.
MA_94930g0010	*PaPB1*	PF00564-PB1 domain	AT3G26510.4	Octicosapeptide/Phox/Bem1p family protein;	Protein binding. Protein interaction data indicates it may participate in signaling pathways alongside other proteins such as the calcium-dependent protein kinase CPK11
MA_99608g0010	*PaGER5*	GRAM domain family protein	AT5G13200.1	GEM-RELATED 5;GER5	Encodes a protein with unknown function that is involved in hormone mediated regulation of seed germination/dormancy, Abscisic acid responsive.
MA_159389g0010	*PaAGO2*	PF02171-Piwi domain	AT1G69440.1	AGO2, ARGONAUTE2, PIWI	Acts as the catalytic engine of the RNA-induced silencing complex (RISC)
MA_10425786g0020	*PaAGO7*	PF02170-PAZ domain;PF08699-Domain of unknown function (DUF1785)	AT1G69440.1	AGO7, ARGONAUTE7, ZIP, ZIPPY	Acts as the catalytic engine of the RNA-induced silencing complex (RISC)
MA_10431106g0020	*PaTGH1a*	PF01805-Surp module; SWAP/Surp superfamily	AT5G23080.2	TGH, TOUGH - RNA binding and processing proteins	It is an important component of miRNA and siRNA biogenesis.
MA_94046g0010	*PaSKIP4*	F-box Kelch-repeat protein		F-box, Kelch_1	F-box Kelch-repeat proteins (KFBs) are large, largely plant-specific gene families involved in targeted protein degradation. By linking specific target proteins to the SCF (SKP-CULLIN-F-BOX) ubiquitin-ligase complex, they act as molecular matchmakers that regulate plant growth, stress responses, flowering time, and the circadian clock
MA_168616g0030	*PaELP1*	elongator complex protein		WD domain, G-beta repeat	The **Elongator complex** in plants (frequently referred to as **ELO/ELP**) is a highly conserved, 6-subunit eukaryotic complex associated with RNA polymerase II. These WD domains act as a scaffold for protein-protein interactions, holding the catalytic subcomplexes together and recognizing target tRNAs. In plants, this specific gene family is deeply involved in morphological development and environmental adaptation
MA_10431337g0020	*PaAMPD1/PaFAC1*	AMP deaminase/Embryonic Factor 1		Adenosine/AMP deaminase	FAC1 is one of the earliest expressed genes in plant embryogenesis. While it is expressed at low levels in mature plant organs, its transcription surges during early zygotic development, endosperm formation, and somatic embryogenesis. Arabidopsis thaliana—encodes an AMP deaminase (AMPD)
MA_10013703g0010	*PaADR1*	disease resistance NB-LRR family		NB-ARC, RPW8	The **ADR1** (Activated Disease Resistance 1) gene family in plants encodes a critical group of **helper NLR** (Nucleotide-Binding Leucine-Rich Repeat) immune receptors.
MA_91728g0010	*PaMAPK1*	mitogen-activated protein kinase		Pkinase	Mitogen-activated protein kinase (MAPK) genes in plants encode highly conserved signaling proteins that regulate vital growth, developmental, and defense processes. Acting via sequential phosphorylation cascades (MAPKKK → MAPKK → MAPK), they transduce extracellular environmental cues into intracellular responses
MA_1408g0010	*PaUPL1*	E3 ubiquitin-protein ligase RF298		Zinc finger, C3HC4 type (RING finger)	The **RF298 gene** in plants (best studied in *Arabidopsis thaliana*) encodes a **RING-finger E3 ubiquitin-protein ligase**. It is a vital post-translational regulator of cellular proteins, playing significant roles in stress responses and hormone signaling (such as ABA signaling), and acts via the ubiquitin-proteasome system (UPS) to target specific proteins for degradation
