# Supplementary material for: Stable and temporally shifted DNA methylation differences between 14-year-old Norway spruce epitypes under common garden conditions
Source: Planta. 2026 Aug 7;264(3):88. doi: 10.1007/s00425-026-05116-6 (PMC13451259; doi:10.1007/s00425-026-05116-6)
Supplement: Supplementary file 2 [file 425_2026_5116_MOESM1_ESM.docx]

**CE**

**WE**

**Suppl. Fig. S1**. Difference in bud burst progress in clonal (clone B10V) Norway spruce CE and WE epitype trees on 26 May 2016 in the epitype trial in the common garden at Hogsmark, Ås, Norway (59°40'07"N 10°43'04"E). The epitype trees originated from somatic epitype embryos developed under 18˚C (CE) or 28˚C (WE).

CG

**Suppl. Fig. S2.** The average DNA methylation levels for each of the DNA methylation contexts CHH, CHG and CpG in 50 bp windows along 2 kb of the promoter and 1 kb of the gene body at either side of the transcriptional start site (TSS) of 2744 targeted genes through the annual development in shoot tips/buds of Norway spruce clonal epitype trees originating from somatic epitype embryos developed at 18°C (cool epitype; CE) or 28°C (warm epitype; WE) compared with the apical (SAM) and basal (RAM) parts of somatic CE and WE embryos of the same clone (B10V; SAM and RAM embryo data from Viejo et al., 2023).

SE

**Suppl. Fig. S3.** Chord plot showing shared differentially methylated genes (CG context) between somatic CE and WE epitype embryos of the clone B10V (SE) and shoot tips/buds of corresponding clonal Norway spruce epitypes trees, originating from somatic embryos of the same clone (developed at 18°C for CE or 28°C for WE). Shoot tip/bud samples were collected at 10 time points (T1 – T10; see Fig. 2 for dates) throughout the annual cycle. SE embryo methylation data are from Viejo et al., 2023).
